# Red light camera interventions for reducing traffic violations and traffic crashes: A systematic review

**DOI:** 10.1002/cl2.1091

**Published:** 2020-06-29

**Authors:** Ellen G. Cohn, Suman Kakar, Chloe Perkins, Rebecca Steinbach, Phil Edwards

**Affiliations:** ^1^ Department of Criminology and Criminal Justice Florida International University Miami Florida; ^2^ London School of Hygiene and Tropical Medicine London UK

## Abstract

**Background:**

Road traffic crashes are a major and increasing cause of injury and death around the world. In 2015, there were almost 6.3 million motor vehicle traffic crashes in the United States. Of these, approximately 1.7 million (27%) involved some form of injury and 32,166 (0.5%) resulted in one or more fatalities (National Highway Traffic Safety Administration, 2016, Traffic Safety Facts 2013: A Compilation of Motor Vehicle Crash Data from the Fatality Analysis Reporting System and the General Estimates System). The most common cause of urban crashes appears to be drivers running red lights or ignoring other traffic controls and injuries occur in 39% of all of these types of crashes (Insurance Institute for Highway Safety, IIHS, 2018, Red light running). While many drivers obey traffic signals, the possibility for violations exists due to issues such as driver distraction, aggressive driving behaviors, or a deliberate decision to ignore the traffic signal. One researcher suggests that eliminating traffic violations could reduce road injury crashes by up to 40% (Zaal, 1994, *Traffic law enforcement: A review of the literature*). Red light cameras (RLCs) are an enforcement mechanism that permit police to remotely enforce traffic signals; they may serve as a deterrent to drivers who intentionally engage in red light running (RLR). The one previous systematic review of RLCs found that they were effective in reducing total casualty crashes but also found that evidence on the effectiveness of cameras on red light violations, total crashes, or specific types of casualty crashes was inconclusive. However, this review searched only a small number of electronic databases and was limited to a handful of studies published in 2002 or earlier.

**Objectives:**

This report updates and expands upon the previous Cochrane systematic review of RLCs. The aim of this review is to systematically review and synthesize the available evidence on the effectiveness of RLCs on the incidence of red light violations and the incidence and severity of various types of traffic crashes.

**Search Methods:**

This study uses a four‐part search strategy that involves: (a) searching 27 online electronic bibliographic databases for published and unpublished evaluations of RLCs; (b) searching the websites of 46 international institutes and research agencies focusing on transportation issues for reports and other gray literature; (c) searching the reference lists of published studies to identify additional published and unpublished works; and (d) conducting a keyword search using Google and Google Scholar to search for additional gray literature.

**Selection Criteria:**

The criteria for inclusion were determined before the search process began. To be eligible, studies must have assessed the impact of RLCs on red light violations and/or traffic crashes. Studies must have employed a quantitative research design that involved randomized controlled trials, quasi‐random controlled trials, a controlled before‐after design, or a controlled interrupted time series. Research that incorporated additional interventions, such as speed cameras or enhanced police enforcement, were excluded, although normal routine traffic enforcement in the nonintervention control condition was not excluded. Both published and unpublished reports were included. Studies were eligible regardless of the country in which they were conducted or the date of publication. Qualitative, observational, or descriptive studies that did not include formal comparisons of treatment and control groups were excluded from this research.

**Data Collection and Analysis:**

Initial searches produced a total of 5,708 references after duplicates were removed. After title and abstract screening, a total of 121 references remained. Full‐text review of these works identified 28 primary studies meeting the inclusion criteria, in addition to the 10 studies identified in the prior Cochrane review. Because several of the primary studies reported on multiple independent study areas, this report evaluates 41 separate analyses. At least two review authors independently assessed all records for eligibility, assessed methodological risk of bias, and extracted data from the full‐text reports; disagreements were resolved by discussion with a third review author. To facilitate comparisons between studies, a standardized summary measure based on relative effects, rather than differences in effects, was defined for each outcome. Summary measures were calculated for all studies when possible. When at least three studies reported the same outcome, the results were pooled in a meta‐analysis. Pooled meta‐analyses were carried out when at least three studies reported the same outcome; otherwise, the results of individual studies were described in a narrative. Heterogeneity among effect estimates was assessed using *χ* ^2^ tests at a 5% level of significance and quantified using the *I* ^2^ statistic. EMMIE framework data were coded using the EPPIE Reviewer database.

**Results:**

The results of this systematic review suggest that RLCs are associated with a statistically significant reduction in crash outcomes, although this varies by type of crash, and suggest a reduction in red light violations. RLCs are associated with a a 20% decrease in total injury crashes, a 24% decrease in right angle crashes and a 29% decrease in right angle injury crashes. Conversely, however, RLCs are also associated with a statistically significant increase in rear end crashes of 19%. There was also some evidence that RLCs were associated with a large reduction in crashes due to red light violations. There is no evidence to suggest that study heterogeneity is consistently explained by either country or risk of bias, nor did the presence or absence of warning signs appear to impact the effectiveness of RLCs. Studies accounting for regression to the mean tend to report more moderate decreases for right angle crashes resulting in injury than studies not accounting for regression to the mean. Studies with better control for confounders reported a nonsignificant decrease in right angle crashes, compared with a significant decrease for all studies.

**Authors' Conclusions:**

The evidence suggests that RLCs may be effective in reducing red light violations and are likely to be effective in reducing some types of traffic crashes, although they also appear linked to an increase in rear end crashes. Several implications for policymakers and practitioners have emerged from this research. The costs and benefits of RLCs must be considered when implementing RLC programs. The potential benefits of a reduction in traffic violations and in some types of injury crashes must be weighed against the increased risk of other crash types. The economic implications of operating an RLC program also must be considered, including the costs of installation and operation as well as the economic impact of RLC effects.

## 1 PLAIN LANGUAGE SUMMARY

### 1.1 Red light cameras (RLCs) reduce injuries but may have no effect on total crashes

RLCs photograph violators at traffic signals. They can reduce red light running (RLR), total injury crashes, and right angle crashes. However, they may also increase the risk of rear end crashes. The impact of RLCs on other types of crashes, including total crashes overall, is unclear.

### 1.2 What is this review about?

Road traffic crashes are a major cause of injury and death around the world. Many crashes occur because drivers run red lights. RLCs photograph violators, and are used to remotely enforce traffic signals as part of strategies to reduce RLR and traffic crashes. However, there are questions about their effectiveness, and there have been a number of legal challenges to their use.

This review integrates findings from 37 controlled before‐after (CBA) studies, and one controlled interrupted time series (ITS) study, that examine the effect of RLCs on RLR and various types of traffic crashes.

**What is the aim of this review?**

This Campbell systematic review examines the effect of red light cameras on red light running and various types of traffic crashes. The review summarizes results from 38 studies covering 41 evaluations, including 37 controlled before‐after studies and one study using a controlled interrupted time series design. The majority of the studies were conducted in the United States or Australia.

### 1.3 What studies are included?

Included studies measure RLC effectiveness by comparing intersections with cameras to those without them. Studies that examined area‐wide programs, in which RLCs operated at some but not all signalized intersections in the community were also included.

Before‐after studies were only included when they had a distinct control group and collected data for treatment and control conditions both before and after RLCs were put into operation. Studies involving additional interventions, such as speed cameras or enhanced police enforcement, were excluded.

This review summarizes 38 studies that contain 41 eligible evaluations of the effects of RLCs on RLR and/or traffic crashes. The studies come from four countries, with the majority carried out in the United States or Australia. Five of the 38 studies were assessed as having a low risk‐of‐bias and eight were assessed as having a moderate risk‐of‐bias.

### 1.4 Do RLCs reduce RLR and traffic crashes?

RLCs are effective at reducing right angle crashes, right angle injury crashes, and total injury crashes. However, they also appear to increase rear end crashes.

There is some indication that RLCs reduce total crashes due to RLR, but this effect was not significant. Additionally, there is some evidence, from three studies, that RLCs may reduce violations.

Other types of crashes did not appear to be significantly affected by the use of RLCs.

The economic implications of implementing RLC programs is not clear as few studies examined this. Overall, the costs of RLC programs tend to outweigh revenue. Studies of the effect of RLC programs on crash costs produced inconsistent results.

The potential benefits of a reduction in traffic violations and in some types of injury crashes should be weighed against the increased risk of other crash types.

### 1.5 What do the findings of this review mean?

Investing community and police resources in RLCs will reduce various types of traffic crashes, including total crashes involving injuries, and may reduce red light violations, but will also increase rear end crashes.

The majority of the studies examined were found to use weak methods which have a risk of bias. Policymakers and practitioners need to use evidence from better quality studies, particularly randomized controlled trials (RCTs) or natural experiments.

More high‐quality empirical studies of RLCs are needed. Future research may be informed by the information reported in this review.

### 1.6 How up‐to‐date is this review?

The authors of this review searched for studies up to June 12, 2015.

## 2 BACKGROUND

### 2.1 The problem, condition, or issue

Road traffic crashes are a major and increasing cause of injury and death around the world, with almost 1.25 million people dying annually and between 20 and 50 million more suffering non‐fatal injuries, including permanent disabilities. Global estimates suggest that these crashes cost countries between 3% and 5% of their gross income (World Health Organization, 2016).

According to the National Highway Traffic Safety Commission (NHTSA, 2016), there were almost 6.3 million motor vehicle traffic crashes in the United States in 2015. Of these, approximately 1.7 million (27%) involved some form of injury and 32,166 (0.5%) resulted in one or more fatalities; the remainder involved only property damage. Only about 50% of traffic crash fatalities were drivers, the remainder were primarily vehicle passengers, motorcyclists, and nonoccupants (including both pedestrians and bicyclists). The annual economic cost of reported and unreported traffic crashes in the United States has been estimated at $242 billion.

According to the Department for Transport (2016), there were approximately 140,000 traffic crashes in Great Britain in 2015. As a result of these crashes, over 1,730 people were killed and over 186,000 were injured. Approximately 60% of those injured and 44% of traffic crash fatalities were drivers or passengers in cars; the remainder were primarily pedestrians, motorcyclists, and pedal cyclists/bicyclists.

Traffic violations occur when a driver enters an intersection after the traffic light has turned red. While many drivers obey traffic signals, the possibility for violations does exist, due to either driver distraction, aggressive driving behaviors, or a deliberate decision to ignore the traffic signal. Traffic light violations appear to be fairly common. According to the Insurance Institute of Highway Safety (IIHS, 2017), the most common type (22%) of crash occurs as a result of drivers running red lights or ignoring other traffic controls. A recent national telephone survey found that while the vast majority of drivers (93.5%) consider RLR to be unacceptable, almost 39% admitted to having driven through a red traffic light in the past month and over 25% had done this more than once, although very few (2.5%) reported running red lights regularly or fairly often (AAA Foundation for Traffic Safety, 2016). In the United Kingdom, a recent survey found that about 25% of motorists admit to running a red light in the past year, which is equivalent to 9.3 million motorists (Massey, 2016). Comparable information regarding the percentage of crashes in the United Kingdom due to RLR was not available.

RLR can have severe consequences when it results in collisions that cause damage to vehicles and road users. While such traffic crashes may cause damage to property only, they can be serious, particularly when colliding at speed into the sides of other vehicles (Transport Research Board, 2003). Drivers of vehicles approaching traffic lights may also decide that they have time to cross on a yellow or amber light, resulting in a rear end collision if the vehicle in front has already slowed and stopped. In the United States, 771 people were killed during 2015 and approximately 137,000 were injured in traffic crashes involving RLR. About half of the fatalities were the drivers who ran the red lights, the other half included pedestrians, bicyclists, and the occupants of vehicles struck by red light runners. Overall, injuries occurred in 39% of all crashes involving motorists who ran traffic controls such as red lights and stop signs (IIHS, 2018). According to Zaal (1994), eliminating traffic violations could reduce road injury crashes by as much as 40%.

### 2.2 The purposes of traffic signals

Intersections are locations on roads that have the potential to create conflict for drivers and pedestrians and increase the risk of crashes. Although intersections make up only a small proportion of the roadway system in the United States, a considerable proportion of crashes occur at intersections (Choi, 2010). One way to reduce this conflict is through the use of a traffic control device such as a traffic signal. Traffic signals are designed to identify which vehicles and/or pedestrians have the right of way to pass through an intersection at any given time, thus ensuring orderly movement of traffic, reducing delays for waiting vehicles, and reducing the frequency of vehicular crashes (Federal Highway Administration [FHA], 2004a). The Federal Highway Administration's *Manual on Uniform Traffic Control Devices* (FHA, 2012) specifically identifies situations where traffic conditions require the installation of traffic signals; generally, these relate to situations where conflicting traffic movements that create crash potential could exist (Bochner & Walden, 2010).

### 2.3 The use of RLCs as an intervention

Motorists run red lights for a variety of reasons. However, survey research suggests that many drivers consider RLR to frequently be an intentional act that has few legal consequences (FHA, 2004b). There are a number of engineering countermeasures that focus on engineering design as a way to reduce RLR. One increasingly popular method of enforcing compliance with traffic signals is through the use of RLCs.

RLCs are a fully automated photo detection system that includes three key elements: cameras, sensors or triggers, and a computer. The cameras may take still or video images, or both; modern systems generally use digital cameras but some older systems may use 35‐mm cameras. They may be located on one arm of an intersection where a RLR problem has been identified or be placed on all four corners of an intersection, so that vehicles coming from any direction may be photographed from multiple angles. Cameras are activated if the vehicle is moving over the triggers at a predetermined speed; if the vehicle has stopped on an induction loop or activates only the first of the two triggers, the computer will not signal the cameras. Most systems take at least two photographs and also superimpose the date and time of the violation, the location of the intersection, the speed at which the vehicle was traveling and the amount of time that elapsed between the light turning red and the vehicle entering the intersection (FHA, 2004b).

After the RLCs capture images of vehicles as they violate a red traffic signal and the evidence is reviewed, penalty tickets are sent to the address where the violating vehicle is registered. RLCs thus have the potential to reduce traffic law offenses by increasing offenders' perceptions of the risks of being caught and being brought to justice if they run a red light.

RLCs permit police to remotely enforce traffic signals. Unlike traditional manual enforcement methods, which are resource intensive and high risk, RLCs operate continuously and without human intervention, freeing up officers to engage in other activities. They do not lead to potentially dangerous high‐speed pursuits and they provide a physical record of all violations (Bochner & Walden, 2010). Their mechanical nature also reduces the possibility of accusations of human bias, discrimination, or selective enforcement (Aeron‐Thomas & Hess, 2005). Studies have shown that drivers who intentionally engage in RLR appear to be most likely to be influenced by countermeasures of this type (FHA, 2004b).

The use of RLCs, however, remains somewhat controversial, particularly in the United States. Some police departments in the United States have had difficulty sustaining the financial viability of RLC programs, there have been a number of legal challenges to the use of RLCs, and their effectiveness in reducing RLR and vehicular crashes has been questioned (Langland‐Orban, Pracht & Large, 2008; IIHS, 2018). In the United Kingdom, RLCs are generally more accepted as bringing about positive road safety benefits with a rapid growth in their numbers since initial use began in the 1990s (see e.g., Hooke, Knox & Portas, 1996).

### 2.4 Prior reviews

One previous Cochrane systematic review of the effect of RLCs on the incidence of red light violations as well as the incidence and severity of road crashes and casualties has been conducted, examining research published in or before 2002 (Aeron‐Thomas & Hess, 2005). Although no randomized controlled studies were located, the review did identify a number of CBA studies. The study concluded that RLCs were effective in reducing the total number of casualty crashes but also found that evidence regarding the effect of RLCs on red light violations, total collisions, or specific types of casualty crashes was inconclusive. The review concluded that larger and better‐controlled studies were needed.

### 2.5 The use of EMMIE within systematic reviews

This review was conducted in support of the efforts of the What Works Centre for Crime Reduction, which is hosted by the UK College of Policing. The What Works Centre emphasizes the development of an evidence‐based approach to policing by coordinating collaborations among academics and practitioners and creating a program to foster systematic reviews of research into policing and crime reduction practices. The Centre focuses on providing “robust and comprehensive evidence that will guide decision‐making on public spending” (College of Policing, 2016).

The results of this review will be incorporated in an online toolkit devised by researchers at the University College of London (UCL) Jill Dando Institute of Security and Crime Science and hosted by the What Works Centre. The toolkit uses the EMMIE framework, which assesses interventions based on five key dimensions: effect, mechanism, moderators, implementation, and economic cost (Johnson, Tilley & Bowers, 2015).

*Effect* refers to the effectiveness or impact of the intervention and assesses whether or not the evidence suggests that the intervention led to a change in crime, either an increase or a decrease. *Mechanism* refers to how the intervention works and what element of the intervention process brought about the effect. *Moderators* are the context in which the intervention works; this dimension considers the conditions or circumstances that must exist for the intervention to be effective. *Implementation* focuses on what must be done to put the intervention into practice. Finally, *Economics* considers both direct and indirect costs associated with the intervention as well as any possible cost benefits to the implementing agency (College of Policing, 2015).

### 2.6 Contribution of this review

This systematic review has expanded and updated the previous Cochrane systematic review (Aeron‐Thomas & Hess, 2005), which only searched a small number of electronic databases and only included a small number of studies published in 2002 or earlier. Since this study was conducted, the use of RLCs has expanded considerably (IIHS, 2020). RLC technology has also continued to improve; for example, new radar technology has been developed that improves the images obtained from the camera and also allows the system to enforce other traffic violations in addition to RLR (Mitchell, 2012). This updated review involves broader and more extensive searches and incorporates more recent research from as many countries as possible, as well as carrying out more detailed and extensive meta‐analyses and examining economic data when available. Additionally, the review has been expanded to include information from the EMMIE framework. The results of this review have the potential to inform the police and influence government policies and procedures intended to increase traffic safety.

## 3 OBJECTIVES

The main objective of this review was to assess the impact of RLCs on the incidence of red light violations and the incidence and severity of traffic crashes by locating and examining all the major empirical studies on the effect of RLCs on traffic patterns. The update has been expanded by including information under the EMMIE framework (see above) on mechanisms, moderators, implementation, and economic costs of RLC interventions. The description of each study includes the setting (e.g., nature of roads), theoretical basis for the intervention, characteristics, and outcomes (including traffic law violations). Where sufficient numbers of well‐designed controlled evaluations were identified, estimates of the effect of interventions on the defined primary outcome (number of red light violations) and secondary outcomes (e.g., road traffic crashes) are included to assess the effectiveness of interventions. In addition to examining the impact of RLCs on road traffic crashes overall, the effect on different types of traffic crashes, such as rear end and right angle crashes, was evaluated separately. This study has also investigated potential moderators of intervention effects, and summarized the different aspects of implementation of traffic enforcement devices and their respective costs.

## 4 METHODS

### 4.1 Criteria for considering studies for this review

#### 4.1.1 Deviations from protocol

Although every effort was made to comply with the original protocol, some changes were deemed to be essential. These primarily occurred because the original protocol was created by the researchers at FIU, before the FIU/LSHTM collaboration developed. After this collaboration began, some modifications to the protocol were necessary to conform to the requirements of the grant that the LSHTM researchers received from the UK College of Policing. Following is a list of deviations from the protocol:

1. The original FIU protocol included a requirement that studies must collect data for a minimum of one year to be eligible for inclusion in the review. This requirement was removed at the request of the LSHTM researchers. However, the majority of the included studies still meet the original requirement.
2. The list of keywords for the agency and gray literature searches was expanded from the original list in the protocol, as additional relevant keywords were identified. The LSHTM search protocol for electronic databases was also added.
3. The original screening and review process was revised to include the incorporation of studies identified by LSHTM and the National Police Library.
4. The use of the EPPI Reviewer 4 software and the EMMIE framework data extraction were incorporated to meet the requirements of the LSHTM agreement with the College of Policing.

#### 4.1.2 Types of studies

To be eligible for inclusion, a study must have measured the effectiveness of RLCs by comparing intersections that received the treatment (the treatment condition) with intersections that did not (the control condition). We identified studies were eligible for inclusion if they involved one of the following research designs, as they were defined in the original review (based on the Cochrane Effective Practice and Organisation of Care group):

1. Experimental design/RCT: This category included studies that used random assignment to assign intersections to the treatment and control groups.
2. Quasi‐random design/quasi‐RCT: This category included studies that allocated the treatment and control conditions using quasi‐random processes, rather than truly randomizing treatment allocation.
3. CBA design: This quasi‐experimental category included studies in which data were collected for both treatment and control conditions before and after the treatment was initiated.
4. Controlled ITS: This quasi‐experimental category included studies in which data were collected at multiple separate time points before and after the treatment was initiated.

Qualitative, observational, or descriptive studies that did not include formal comparisons of treatment and control groups were excluded from this research.

For all research designs, the nonintervention control condition did not exclude normal routine traffic enforcement by criminal justice system personnel. Police still could issue citations for traffic violations at control intersections during the study period.

#### 4.1.3 Types of participants

RLC studies do not have participants in the standard sense. Essentially, the participants were signalized intersections in the area under study. Intersections where additional interventions were in operation, such as speed cameras or enhanced police enforcement, were excluded. For treatment intersections, RLC enforcement must have applied to all motorists passing through the intersections where the cameras were installed.

#### 4.1.4 Types of interventions

Eligible studies must have tested the effect of RLCs on traffic red light violations or crashes. An RLC is considered to be a digital or film still and/or video camera that is used to detect red light violators and identify them so that they may be charged with their violations. Studies that examined RLCs as part of a larger traffic enforcement initiative, specifically those that examined the joint effect of red light and speed cameras, were excluded.

Studies were included when the interventions included cameras at intersections or junctions that were designed to detect red light violators. Studies examining area‐wide programs where RLCs operated at some but not all signalized intersections in the community were also included.

#### 4.1.5 Types of outcome measures

Eligible studies had to have measured and reported data on at least one of the following outcome measures:

- Red light violations, based on the number of vehicles passing through a junction after entering on a red light. Vehicles that enter a junction on a yellow/amber light but do not clear the intersection before the light changes to red are not considered to be violators.
- Number, severity, and type of road traffic crashes. This may include the number of total crashes, the number of crashes resulting in injury, the number of property damage‐only (PDO) crashes, and the number of specific types of crashes (e.g., rear end crashes; right angle crashes).

Data on economic outcomes, including the costs of providing the intervention and the income it generated, and process outcomes (e.g., implementation data) were also collected when available.

#### 4.1.6 Types of settings

Studies were eligible regardless of the country in which they were conducted or the form in which they were published. When studies were not published in English, efforts were made to obtain translations. There was only one possibly‐eligible study for which this was not possible (Giæver & Tveit, 1998).

No date restrictions were placed on this study. However, RLCs have only been used for traffic enforcement since the 1960s (Retting, Ferguson, & Hakkert, 2003); therefore, no research prior to that time could have been conducted.

### 4.2 Search methods for identification of studies

#### 4.2.1 Search strategy

A four‐part search strategy was used to locate research meeting the criteria for eligibility:

1. Online electronic bibliographic databases were searched for published evaluations of RLCs (see Appendix A for a list of electronic databases).
2. The websites of a large number of international institutes and research agencies focusing on transportation issues were searched for reports and other gray literature (see Appendix B for a list of websites).
3. The reference lists of published studies were searched to identify additional published and unpublished research studies.
4. A keyword search using Google and Google Scholar was conducted to search for additional gray literature. The first 100 nonsponsored hits of each search were examined.

#### 4.2.2 Electronic searches

For all agency website and gray literature searches, the following keywords were used:

- Red AND light AND camera(s)
- Red light AND camera(s)
- Red AND light AND violation(s)
- Red light AND violation(s)
- Traffic AND camera(s)
- Traffic AND violation(s)
- Traffic AND light(s)

These were adapted when necessary to meet specific requirements of individual search engines or to conform to international terminology variations and spelling conventions. Search terms were intentionally general in nature to ensure that searches cast the broadest possible net and that relevant background material was also identified.

All electronic database searches were conducted using the full search strategy as outlined in Appendix C and only superficially adapted for each database. Additional specialized searching was conducted by the College of Policing's National Police Library.

### 4.3 Data collection and analysis

#### 4.3.1 Screening and review process

All studies identified through the LSHTM and National Police Library search process were first exported to the EndNote bibliographic database for de‐duplication. Once duplicate records had been removed, records were combined with FIU search results in a spreadsheet to identify and remove further duplications. Once duplicate records were removed, each study was screened to determine if it met basic inclusion criteria, specifically:

1. The study dealt with the use of RLCs to reduce/prevent traffic light violations and/or traffic crashes.
2. The study included both a treatment and a comparison/control group.
3. The study reported results on at least one of the following outcome measures: incidence of red light violations, incidence of road traffic crashes, and severity of road traffic crashes.
4. Extraneous variables were controlled by at least one of the following methods: randomization, matching, or pre‐test and post‐test measures of violations and/or crashes.

At least two review authors independently examined the titles, abstracts, and keywords of electronic records for eligibility according to the inclusion criteria above. Results of this initial screening were cross‐referenced between the authors and full‐texts were then obtained for all potentially relevant reports of studies. The publication status of the study (unpublished vs. published) did not affect study eligibility.

The full‐text reports of potentially eligible studies were independently assessed for final inclusion in the review by two review authors using screening codes in EPPI Reviewer 4. Any disagreements were resolved by discussion with a third review author. Reference lists of all eligible trials were inspected for further eligible studies.

#### 4.3.2 Data extraction and management

All studies were managed using the EPPI Reviewer 4 software. For the EMMIE framework data extraction, data were coded independently by two review authors in EPPI Reviewer, using a standardized data coding set (see Appendix D: EPPI Reviewer standardized data coding set for EMMIE framework). The remaining coding of studies (including study characteristics, risk of bias, measurement of effect) was conducted using Microsoft Excel.

#### 4.3.3 Details of study coding categories

All eligible studies were coded on a variety of criteria, including details related to the source of the study (publication source, title, authors, etc.), study characteristics (methodological type, dates of data collection, etc.), sample characteristics (size, location, etc.) study methods and procedures (selection process, characteristics of treatment and control areas, associated publicity campaigns, etc.), descriptions of the independent and dependent variables (construct, operationalization, etc.), effect size data (if any), adjustment for bias (regression to the mean [RTM], spillover/diffusion, etc.), and study conclusions.

Every eligible study was coded by two review authors, using a standardized data extraction instrument (see Appendix E). All disagreements were identified and resolved by discussion with a third review author.

#### 4.3.4 Descriptive analyses

The review includes all studies meeting the inclusion criteria. Descriptive statistics extracted from each study included:

- Study design: This describes study design; risk of bias; data collection methods, types of statistical analyses
- Participants: This describes intervention and controls; setting of study; nature of roads used
- Program components: This describes type of camera; camera signing practices; publicity campaigns
- Study outcomes: This describes incidence of red light violations and traffic crashes

#### 4.3.5 Statistical analyses

To facilitate comparisons of studies, a standardized summary measure was defined for each outcome. Summary measures were based on relative effects, rather than differences in effects, where the outcome after intervention was divided by the outcome before intervention, as an expression of the proportional change in outcome. Summary measures were calculated for all studies where possible (i.e., where required information was reported or adequate data were available for the calculation).

Rate ratios were estimated by dividing the count of the outcome post‐ and preintervention in the intervention area by the corresponding ratio in the control area. For example, the estimated rate ratio for road traffic collisions was:

$$\frac{c\text{ollisions}\;\text{after}/c\text{ollisions}\;\text{before}\;\text{in}\;\text{intervention}\;\text{area}}{c\text{ollisions}\;\text{after}/c\text{ollisions}\;\text{before}\;\text{in}\;\text{control}\;\text{area}}.$$

Assuming that traffic volume remains the same on the roads postintervention in the control and intervention areas, this rate ratio estimates the change in the collision rate in intervention areas compared to that in control areas. For outcomes expressed as counts or rates the intervention effect was estimated using rate ratios with a 95% confidence interval (CI), calculated assuming a Poisson distribution for the number of collisions in each area and time period (however, the adequacy of the fit of the Poisson distribution was not assessed). Where studies provided Empirical Bayes estimates, these were extracted directly from the primary studies with the standard errors (*SE*s), and included in the meta analysis. Similarly for ITS, the coefficient and the *SE* from studies were extracted and used to generate rate ratios and CIs. Studies from which *SE*s could not be extracted (or from which *SE*s were incalculable) were excluded from the final statistical analyses, and the results were described and compared where appropriate.

#### 4.3.6 Data synthesis

Results were pooled in a meta‐analysis where three or more studies reported the same outcome (Cooper, 2003). The logarithm of the rate ratio and its *SE* (calculated assuming a Poisson distribution for the number of collisions in each area and time period) were pooled. If there were too few studies for a meta‐analysis, the results of individual studies were described in a narrative.

Heterogeneity (variability) among the effect estimates was assessed using a *χ* ^2^ test at a 5% significance level and quantified using the *I* ^2^ statistic, the percentage of between‐study variability that is due to true differences between studies (heterogeneity) rather than due than to sampling error. An *I* ^2^ value greater than 50% was considered to reflect substantial heterogeneity. Substantial heterogeneity would mean that the results of different studies vary substantially more than would be expected if the effects of RLCs were the same in each setting. Where substantial variation in results was identified, subgroup analyses were conducted in an attempt to investigate the source. Stratification of the outcome data was only possible where sufficient information on studies was available for grouping. Subgroup analysis was conducted on the basis of risk of bias and by country (United States, Australia, or other). When assessing for differences of effect by subgroups, visual inspection of the forest plots was made and consideration to the widths of the CIs for each subgroup was given. Where the CIs of summary estimates for subgroups do not overlap, or do so very little, a difference in effect between the groups may be indicated, and follow‐up tests of group difference were conducted. Details of each intervention are presented in tables of the characteristics of studies (Appendix F). Stata statistical software (version 14.2) was used to conduct the meta‐analyses.

#### 4.3.7 Assessment of risk of bias in included studies

##### Spillover (diffusion of benefits)

Spillover occurs when the treatment has an effect outside the targeted area or population. In the case of traffic enforcement, spillover occurs when a safety measure such as an RLC that is placed at one intersection affects driver behavior at other intersections that do not have RLCs. This may occur because RLC programs frequently involve not only the placement of cameras but also the posting of warning signs and widespread publicity campaigns. As a result, driver behavior may be affected throughout the area, rather than just at those intersections that have cameras. To reduce spillover effects, control and comparison sites should be located outside the area affected by RLC program publicity.

##### RTM

RTM refers to a statistical phenomenon that appears when making repeated measurements of the same variable. In general, observations that produce extremely high or low values tend to be followed by values that are closer to the mean (see e.g., Barnett, van der Pols, & Dobson, 2005). In the case of traffic enforcement research, safety measures such as RLCs tend to be placed where they can be most effective, which means that they usually are located at intersections with very high rates of traffic violations and/or traffic crashes. These intersections are likely to show lower rates of violations and/or crashes upon later measurement, regardless of whether or not RLCs have been installed, due to the tendency of RTM.

In experimental studies, the effect of RTM can be controlled at the study design stage by randomly allocating subjects (intersections) to treatment or control groups. However, for observational and quasi‐experimental study types RTM can be reduced at the analysis stage by using statistical methods such as random effect (RE) models which produce Empirical Bayes estimates of effects. The RE models calculate an overall effect size by pooling individual observed study effects that account for the variability within and between studies. The individual study Empirical Bayes estimates of effects are then calculated as a weighted average of the observed effect size and the overall effect size estimated from the model. This results in observed effects being "shrunk" toward the overall effect estimate where the degree of "shrinkage" depends on the precision (*SE*) of the observed effect size. Thus, observed effect sizes that are poorly estimated are weighted more heavily toward the overall effect estimate. Empirical Bayes estimates tend to moderate the effect of very large observed effect sizes which may result from RTM.

##### Risk of bias assessment

The expanded risk of bias analysis was based on six dimensions that focused on the design of the study, the analysis of the data, and the contents of the study report. These six dimensions, which conform to the requirements set forth by the UK Economic and Social Research Council (ESRC), are:

1. Selection and matching of intervention and control areas
2. Blinding of data collection and analysis
3. Pre‐ and postintervention data collection periods
4. Reporting of results
5. Control of confounders
6. Control of other potential sources of bias

See Appendix G for a list of the 17 specific criteria included in each dimension. Each individual criterion statement was scored on whether it was True, False, or Unclear and these were used to assess each study on whether it presented a high, low, or unclear risk of bias across the six domains.

Risk of bias assessment was performed independently by three review authors (E. G. C., S. K., and C. P.). For the studies identified in the previous review, the same three review authors independently assessed the risk of bias of the included studies. Any discrepancies were resolved by deferment to further review authors (R. S. and P. E.). All disagreements were resolved by consensus.

## 5 RESULTS

This section presents the results of the systematic review and meta‐analysis of studies examining RLCs. It is organized around the EMMIE framework which includes measures of effect as well as discussions of the mechanisms through which RLCs are believed to work, the moderating factors that may influence the activation of these mechanisms, various elements that may affect the successful implementation of RLC programs, and the economic costs and benefits associated with the use of RLCs.

### 5.1 Description of studies

#### 5.1.1 Results of the search

The search strategy produced a total of 5,708 records after duplicates were removed. Title and abstract screening resulted in the exclusion of 5,587 records, leaving a total of 121 references that were potentially eligible for inclusion in this study. Full‐text review of these works identified 28 primary studies that met the inclusion criteria, in addition to the 10 studies that had been identified in the prior Cochrane review. Eight of these newly identified studies (and one study from the previous review) had associated publications, which were subsumed in the primary studies (Cunningham & Hummer, 2010; Fitzsimmons, 2007a, 2007b, 2007c; Garber, Miller, Abel, Eslambolchi, & Korukonda, 2007; Hallmark, Orellana, McDonald, Fitzsimmons, & Matulac, 2010; Miller, Khandelwal, & Garber, 2006; Persaud, Council, Lyon, Eccles, & Griffith, 2005; Sayed & de Leur, 2007; Shin & Washington, 2007; Retting & Kyrychenko, 2002 from the previous review). Two of the newly identified studies reported on more than one independent study area: Fitzsimmons (2007a,b,c) and Hallmark et al. (2010) included separate analyses of RLCs in two cities in Iowa (Council Bluffs and Davenport) and Shin and Washington (2007) evaluated independent programs in two cities in Arizona (Phoenix and Scottsdale). Kloeden, Edwards, and McLean (2009) evaluated two RLC programs in Adelaide that occurred at different times (1988 and 2001).

The full‐text reports for the 28 newly identified studies (along with their subsumed publications) were coded in detail in EPPI Reviewer and Excel for the EMMIE analysis and are included in the tables of characteristics of included studies (Appendix F). The previous Cochrane review (Aeron‐Thomas & Hess, 2005) applied a naming convention combining authors and study location to the 10 included studies. For the additional studies identified in this updated review, first author names and years of publication have been retained to identify each study, with a city or year suffix where required. From the 28 additional primary studies, there were 31 separate analyses evaluated in this review.

**Figure 1:**
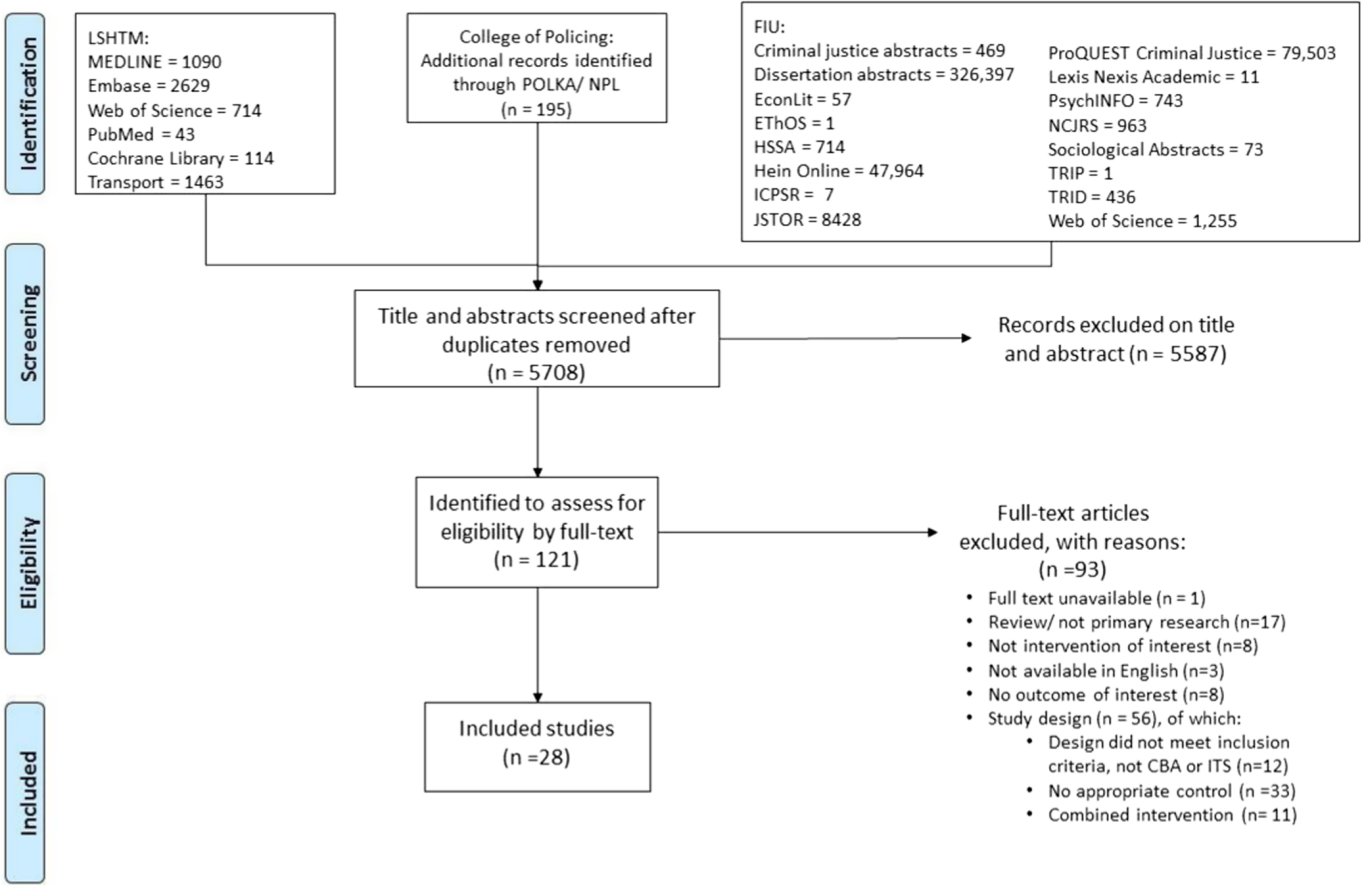
PRISMA flow diagram

The search process is diagrammed in Figure 1, which also shows the number of records excluded, with a summary of reasons. Further details of some of the excluded studies are also available in Appendix F.

#### 5.1.2 Description of included studies

A total of 38 studies were included in this analysis. Of these, 37 were CBA studies with a distinct control group (27 newly identified studies plus 10 previously identified in the prior Cochrane review). One of the newly identified studies employed a controlled ITS design. No RCTs were found. See Appendix F for information on the studies included in and excluded from this review. The characteristics of the studies in the previously published Cochrane systematic review are not duplicated in this report (for copyright reasons).

Like the previous review, the majority of the newly identified studies were from the United States (20) and Australia (5); the remainder were from Canada (2) and Singapore (1). Fifteen of the newly identified studies were published in academic journals (although seven also had other associated publications); the rest were published as technical reports and/or Master's theses. The publication dates of the newly identified studies ranged from 1981 to 2016 inclusively; two studies (Andreassen, 1995; Maisey, 1981) included data from the 1970s. None of the original or newly identified studies distinguished between types of motorists; any vehicle passing through an intersection under study was included in the analyses. The methods of analysis used by study authors varied greatly. Most studies used simple ratios (as we have proposed in this review), while 12 included Empirical Bayes analyses.

The outcome measures from the 38 primary studies covered a range of definitions and measures of red light violations, crashes, and injuries. Only three studies reported red light violations and these studies reported no other outcomes (Arup Transportation Planning, 1992; Chin, 1989; Retting, Williams,, Farmer, & Feldman, 1999a). The rest examined various types of crashes. Most studies reported crash outcomes by crash type and collected crash statistics from official databases of police reports. The types of crashes examined included total crashes (all types combined), rear end crashes, and right angle crashes (or similar outcomes such as turning same roadway crashes; turning different roadway crashes; angle crashes; and turning and right angle crashes) In many studies, crash types were further disaggregated into total number of crashes of a given type (including those involving PDO), injury crashes, and RLR only crashes (identified through police reports where a red light violation occurred).

Fourteen of the newly identified studies reported crash outcomes resulting in injury to passengers or other road users (AECOM Canada, Ltd., 2014; Ahmed & Abdel‐Aty, 2015; Burkey & Obeng, 2004; City of Lubbock, 2008; Cunningham & Hummer, 2010; Garber, 2007; Kloeden et al., 2009; Kull, 2014; Llau, Ahmed, Khan, Cevallos, & Pekovic, 2015; Maisey, 1981; Miller et al., 2006; Sayed & de Leur, 2007; Sharpnack, 2009) as did five of the previously identified studies (Hillier, Ronczka, & Schnerring, 1993; Mann, Brown, & Coxon, 1994; Ng, Wong, & Lum, 1997; Retting & Kyrychenko, 2002; South, Harrison, Portans, & King, 1988). In most cases, injury crashes included total injuries and fatalities. However, Llau et al. (2015) included “possible injuries” in the injury crash counts while Persaud et al. (2005) specifically excluded crashes classified as “possible injury” from the injury class counts. Burkey and Obeng (2004) examined injury and possible injury crashes separately.

Eight of the included studies reported results from which *SE*s could not be calculated: seven newly identified studies (Andreassen 1995; Chin 1989; Kull 2014; Pulugurtha & Otturu, 2014; Richardson 2003; Ross & Sperley, 2011; Sayed & de Leur, 2007) and one original study (South et al., 1988). Seven of these studies were excluded from all final meta‐analyses. South et al. (1988) was included in the final meta‐analyses for crash types where *SE*s could be calculated (total injury crashes, right angle injury crashes and rear end injury crashes) and was excluded from the crash type where this was not possible (turning, same roadway injury crashes).

A summary of outcome measures covered by the current and previous reviews is provided in Table 1.

**Table 1:** Summary of outcome measures

	Number of studies reporting	
Outcome	Current review	Previous review
Total crashes (inc PDO)		
Total	17	7
Right angle	13	2
Turning, same roadway	5	0
Turning, different roadway	1	0
Turning and right angle	3	0
Rear end	17	2
Property damage only	5	2
Violations	2	1
Hit pedestrian	2	0
Injury outcomes		
Total	15	5
Right angle	4	3
Turning, same roadway	2	1
Turning, different roadway	0	0
Turning and right angle	1	0
Rear end	4	3
Red light running		
Red light running	6	0
Red light running right angle	1	0
Red light running rear end	3	0
Red light running injury crashes	2	0

### 5.2 Risk of bias in included studies

Five of the 38 studies received a low risk of bias assessment across all six domains and also accounted for RTM and spillover (all five were newly identified studies: Ahmed & Abdel‐Aty, 2015; Cunningham & Hummer, 2010; Garber, 2007; Llau et al., 2015; Pulugurtha & Otturu, 2014), five received a low risk of bias (including accounting for RTM and spillover) in all but one domain (AECOM Canada, Ltd., 2014; Burkey & Obeng, 2004; Ko, Geedipally, & Walden, 2013; Miller et al., 2006; Sayed & de Leur, 2007) and 28 received a high risk of bias across more than one domain (18 newly identified studies plus 10 previously identified). Table 2 provides a list of the included studies with their risk of bias assessments. Studies were stratified across each of these domains for additional sensitivity analyses.

**Table 2:** Summary of included studies and quality of evidence

Study	Risk of bias						Study design	Spillover	RTM
	Intervention and control	Blinding	Study length	Reporting	Con‐founders	Other bias			
AECOM Canada, Ltd. (2014)	Low	Low	Low	High	Low	Low	CBA, EB	Yes	Yes
Ahmed and Abdel‐Aty (2015)	Low	Low	Low	Low	Low	Low	CBA, EB	Yes	Yes
Andreassen (1995)^a^	Low	Low	High	High	High	Low	CBA	Yes	No
Arup Transportation Planning (1992)	High	High	Low	High	High	High	CBA	No	No
Burkey and Obeng (2004)	Low	Low	Low	Low	Low	Low	ITS	No	Yes
Chin (1989)^a^	High	High	Low	High	High	High	CBA	Yes	No
City of Garland (2006)	Unclear	Low	High	High	High	Unclear	CBA	No	No
City of Lubbock (2008)	Unclear	Low	High	High	High	Unclear	CBA	No	No
Cunningham and Hummer (2010)	Low	Low	Low	Low	Low	Low	CBA	Yes	Yes
Fitzsimmons (2007b)									
Council Bluffs	Low	Low	Low	Low	High	Low	CBA	No	No
Davenport	Low	Low	Low	Low	High	Low	CBA, EB	No	Yes
Garber (2007)	Low	Low	Low	Low	Low	Low	CBA, EB	Yes	Yes
Hobeika and Yaungyai (2006)	Low	Low	High	High	High	Low	CBA	Yes	No
Hu et al. (2011)							CBA	Yes	Yes
Kloeden et al. (2009)									
1988	Unclear	Low	Low	High	High	Unclear	CBA	No	No
2001	Unclear	Low	Low	High	High	Unclear	CBA	No	No
Ko et al. (2013)	Low	Low	Low	High	Low	Low	CBA, EB	Yes	Yes
Kull (2014)^a^	Low	Low	Low	High	High	Low	CBA	Yes	No
Llau et al. (2015)	Low	Low	Low	Low	Low	Low	CBA, EB	Yes	Yes
Maisey (1981)	Low	Low	Low	High	High	Low	CBA	Unclear	No
Miller et al. (2006)	Low	Low	Low	Low	Low	Low	CBA, EB	No	Yes
Persaud et al. (2005)	Low	Unclear	Low	High	Low	Low	CBA, EB	Yes	Yes
Porter et al. (2013)	High	High	High	High	High	High	CBA	Yes	No
Pulugurtha and Otturu (2014)^a^	Low	Low	Low	Low	Low	Low	CBA, EB	Yes	Yes
Richardson (2003)^a^	Low	Low	High	High	High	Low	CBA, EB	No	Yes
Ross and Sperley (2011)^a^	Low	Low	High	High	Low	Low	CBA	No	No
Sayed and de Leur (2007)^a^	Low	Low	Low	Unclear	Low	Low	CBA, EB	Yes	Yes
Sharpnack (2009)	Low	Low	High	High	High	Low	CBA	No	No
Shin and Washington (2007)									
Scottsdale	Low	Unclear	Low	Low	Low	Low	CBA, EB	Yes	Yes
Phoenix	Unclear	Low	Unclear	Low	Low	Low	CBA	Yes	Yes
Sohn and Bandini (2012)	Low	Low	Low	Low	Unclear	High	CBA	No	No
*Previously identified studies*									
CA SA LA 2002	High	Low	Low	High	Unclear	High	CBA	No	No
CA SA Oxnard 2002	High	Low	Low	High	Unclear	High	CBA	No	No
CA SA Sacramento 2002	High	Low	Low	High	Unclear	High	CBA	No	No
CA SA San Diego 2002	High	Low	Low	High	Unclear	High	CBA	No	No
Hillier Sydney 1993	Low	Unclear	Low	High	Low	High	CBA	No	Yes
Mann Adelaide 1994	Unclear	Unclear	Unclear	Unclear	Unclear	High	CBA	No	No
Ng Singapore 1997	Unclear	Low	Low	Low	Low	Low	CBA	No	Yes
Retting Fairfax 1999 (1999a)	Unclear	High	High	Low	Unclear	High	CBA	Yes	No
Retting Oxnard 2002	Low	Low	Low	Low	High	Low	CBA	No	Yes
South Melbourne 1988^b^	Low	Low	Low	Low	High	High	CBA	No	Yes

Abbreviations: CBA, controlled before/after; EB, Empirical Bayes; ITS, interrupted time series; RTM, regression to the mean; *SE*, standard error.

^a^ These seven studies were excluded from final statistical analyses as *SE*s were incalculable.

^b^ This study was excluded from final statistical analyses for turning, same roadway crashes where *SE* was incalculable. It was included for total injury crashes, right angle injury crashes, and rear end injury crashes where *SE* could be extracted.

### 5.3 Effects of interventions

#### 5.3.1 Total crashes

##### Total crashes

Twenty‐four studies reported 27 estimates of effect on the total number of all types of crashes (including PDO). Twenty of these studies (accounting for 23 estimates of effect) reported these estimates with CIs or *SE*s.

**Figure 2:**
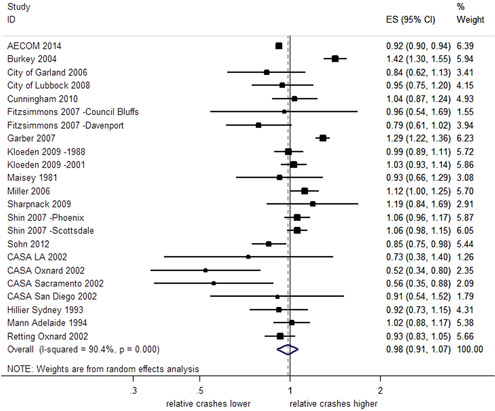
Effects of red light cameras on total crashes. CI, confidence interval; ES, effect size

The effects of RLCs on the total number of crashes at intersections were highly heterogeneous (*I* ^2^ = 90.4%, *Q *= 228.4, and *df* = 22, *p *< .001). The direction of the estimated effects was also inconsistent. Figure 2 shows the effect sizes (ES) and 95% CI for each individual study. When the CI overlaps the reference line (a vertical line located at one on the *x*‐axis), this indicates that the observed effects of RLCs may be due to chance. An overall estimate of the relative change in total crashes at locations with RLCs compared with control sites is represented by a diamond shape at the bottom of Figure 2. This pooled effect estimate of 0.98 indicates a 2% reduction in total crashes. However, as the 95% CI crosses the reference line, ranging from a 9% reduction to a 7% increase in total crashes, the effect is uncertain.

Four newly identified studies (accounting for four estimates of effect) reported results from which *SE*s could not be obtained (Pulugurtha & Otturu, 2014; Richardson 2003; Ross & Sperley, 2011; Sayed & de Leur, 2007). Pulugurtha and Otturu (2014) reported an EB analysis that found the RLC program was not effective in reducing crashes at the majority of selected signalized intersections. This study found some evidence that the RLCs were more effective at reducing total crashes at intersections with less than 40,000 vehicles entering per day, but reported more uncertain and marginal effects at intersections with greater than 40,000 vehicles per day. Using EB analysis to estimate the effect on total crashes, Richardson (2003) reported a reduction of 0.65 crashes per site per year as a result of RLC installation which was equated to an 11.7% reduction in total crashes per year. A before and after study conducted on a single RLC camera site (Ross & Sperley, 2011) reported an increase in total crashes after camera installation, around 77% increase in average number of total crashes per month. Sayed and de Leur (2007) conducted an EB analysis, reporting reductions in total crashes at 20 of the 25 locations after RLC implementation and a reduction in total crashes of 11.1%. These results are aligned with the meta‐analysis which found the effects on total crashes to be heterogeneous, and inconsistent in the estimated direction of effect. Some of these additional studies highlighted variation within crash and intersection types impacting the effect of RLCs.

##### Total crashes stratified by country

**Figure 3:**
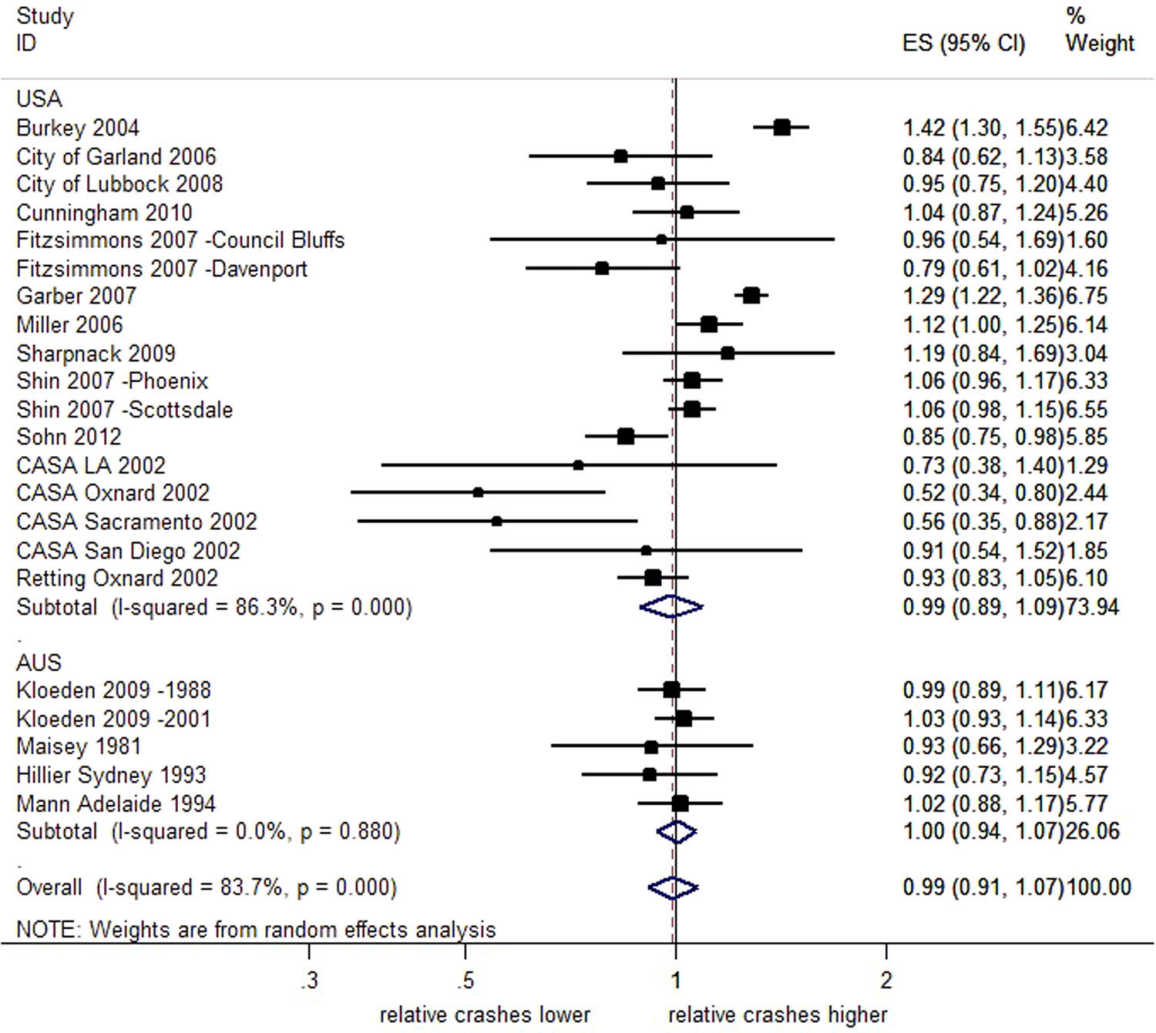
Effects of red light cameras on total crashes—stratified by country. CI, confidence interval; ES, effect size

As shown in Figure 3, the subgroup analysis by country indicates uncertain effects of RLCs on total crashes in both Australia (2% decrease; 95% CI; 6% decrease 7% increase) and the United States (1% decrease; 95% CI; 11% decrease–9% increase). In both cases, the CIs overlap the reference line and a further test of group differences indicates no evidence of difference in the subgroup effects (*Q *= 0.10, *p *= .76). In Australia, the effect estimates across studies are consistent (*I* ^2^ = 0%, *p *< .001) while in the United States, there was much greater heterogeneity (*I* ^2^ = 86.3%, *p* < .001).

##### Total injury crashes

**Figure 4:**
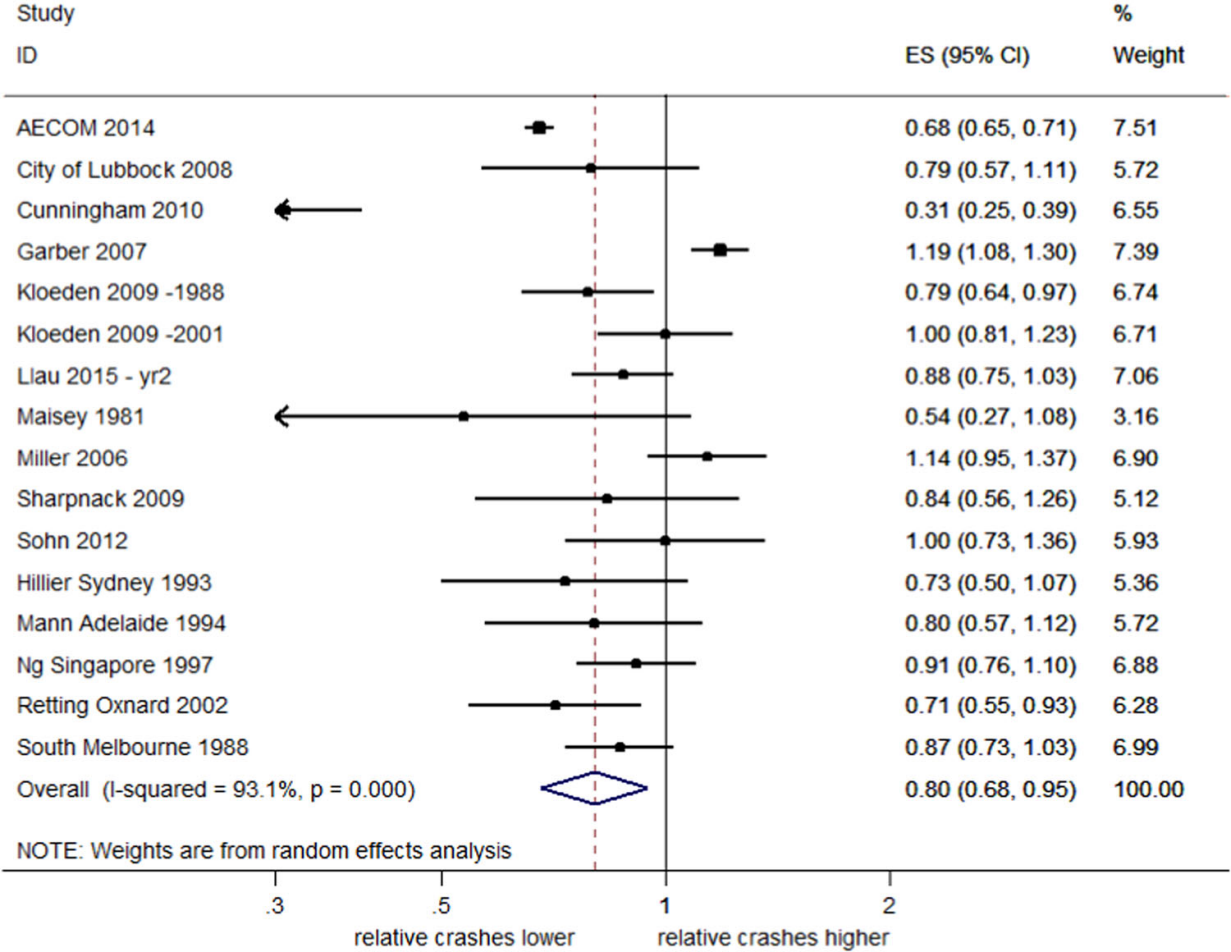
Effects of red light cameras on total injury crashes. CI, confidence interval; ES, effect size

Seventeen studies examined injury crashes, providing 18 estimates of effect. Fifteen of these studies (16 estimates) reported CIs. Figure 4 shows that the estimates of effect on total injury crashes were also highly heterogeneous (*I* ^2^ = 93.1%, *p *< .001). The overall pooled estimate of effect suggests that RLCs reduced total injury crashes by 20% with a 95% CI (32–5% decrease). In Llau et al. (2015), the number of injury crashes specifically included possible injuries; this distinction was not made for other studies.

Two newly identified studies (Kull, 2014; Sayed & de Leur, 2007) reported results from which *SE*s could not be obtained, but reported reductions similar to studies in the meta‐analysis. Kull (2014) reported a reduction in the rate of all injury crashes of 0.48 per intersection per year, while Sayed and de Leur (2007) reported reductions in injury crashes of 6.1% after RLC implementation.

Several other studies used different criteria to measure injuries and therefore are not included in the pooled analysis. Burkey and Obeng (2004) reported increases in crashes resulting in severe injuries (10% increase; 95 CI %; 20% decrease–50% increase), and increases for possible injuries (50% increase; 95% CI; 30–70% increase). Hu, McCartt and Teoh (2011) reported a reduction in all fatal crashes at RLC intersections per million population of 17% (95% CI; 30–0% decrease). Richardson (2003) reported a 29.2% decrease in the number of people injured at red light intersections per year (CI incalculable).

##### Total injury crashes stratified by country

**Figure 5:**
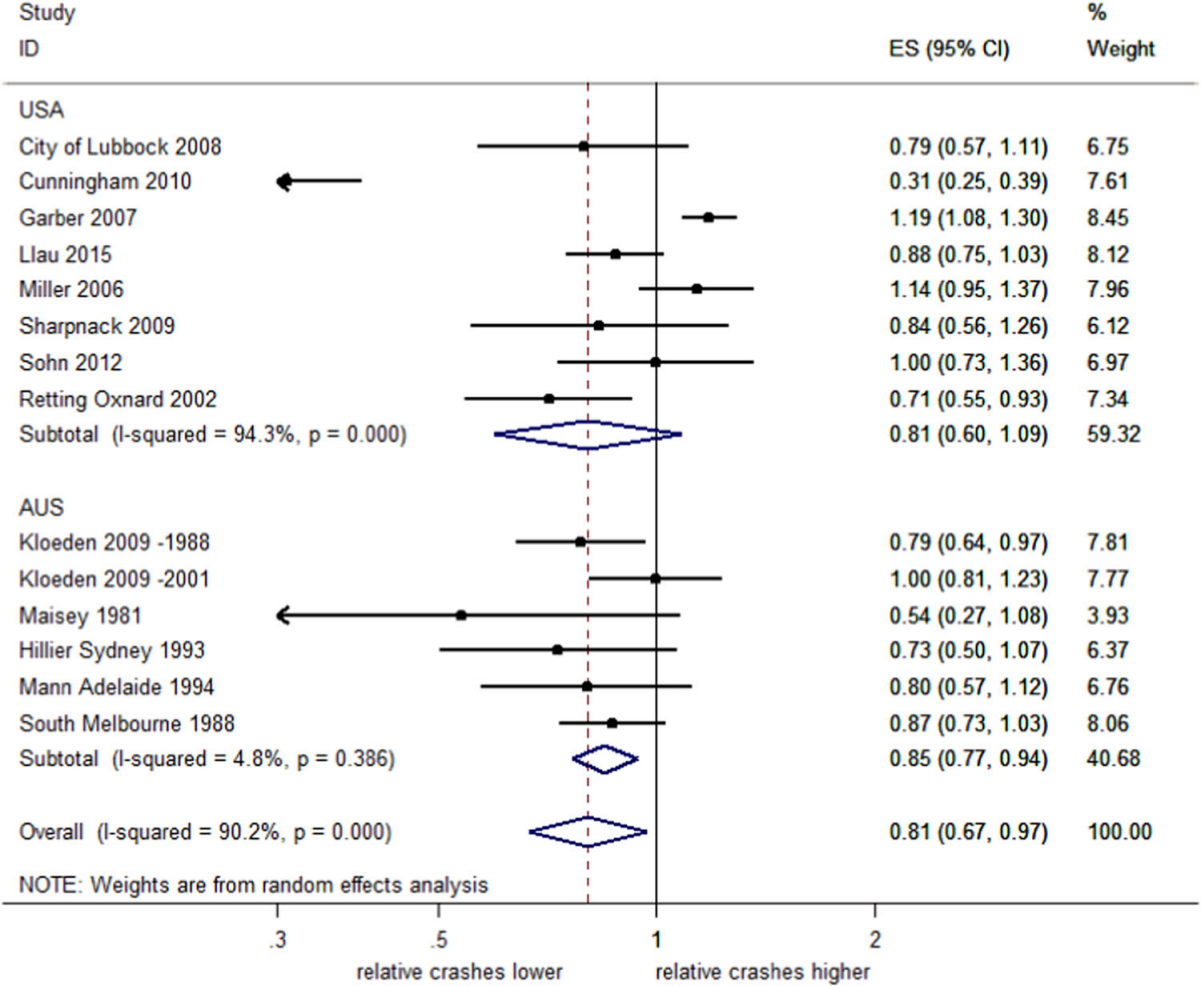
Effects of red light cameras on total injury crashes—stratified by country. CI, confidence interval; ES, effect size

As seen in Figure 5, a meta‐analysis of studies from the United States reported a decrease in injury crashes of 19%, although the effect was uncertain (95% CI; 40% decrease–9% increase) and there was evidence of significant heterogeneity (*I* ^2^ = 94.3%, *p *< 0.001). A meta‐analysis of studies from Australia reported a decrease in injury crashes of 15% (95% CI; 23–6% decrease) with no evidence of heterogeneity (*I* ^2^ = 4.8%, *p *< .001). The test of group differences indicates no evidence against the null hypothesis that the subgroup effects are the same (*Q *= 0.11, *p *= .74).

##### Total PDO crashes

**Figure 6:**
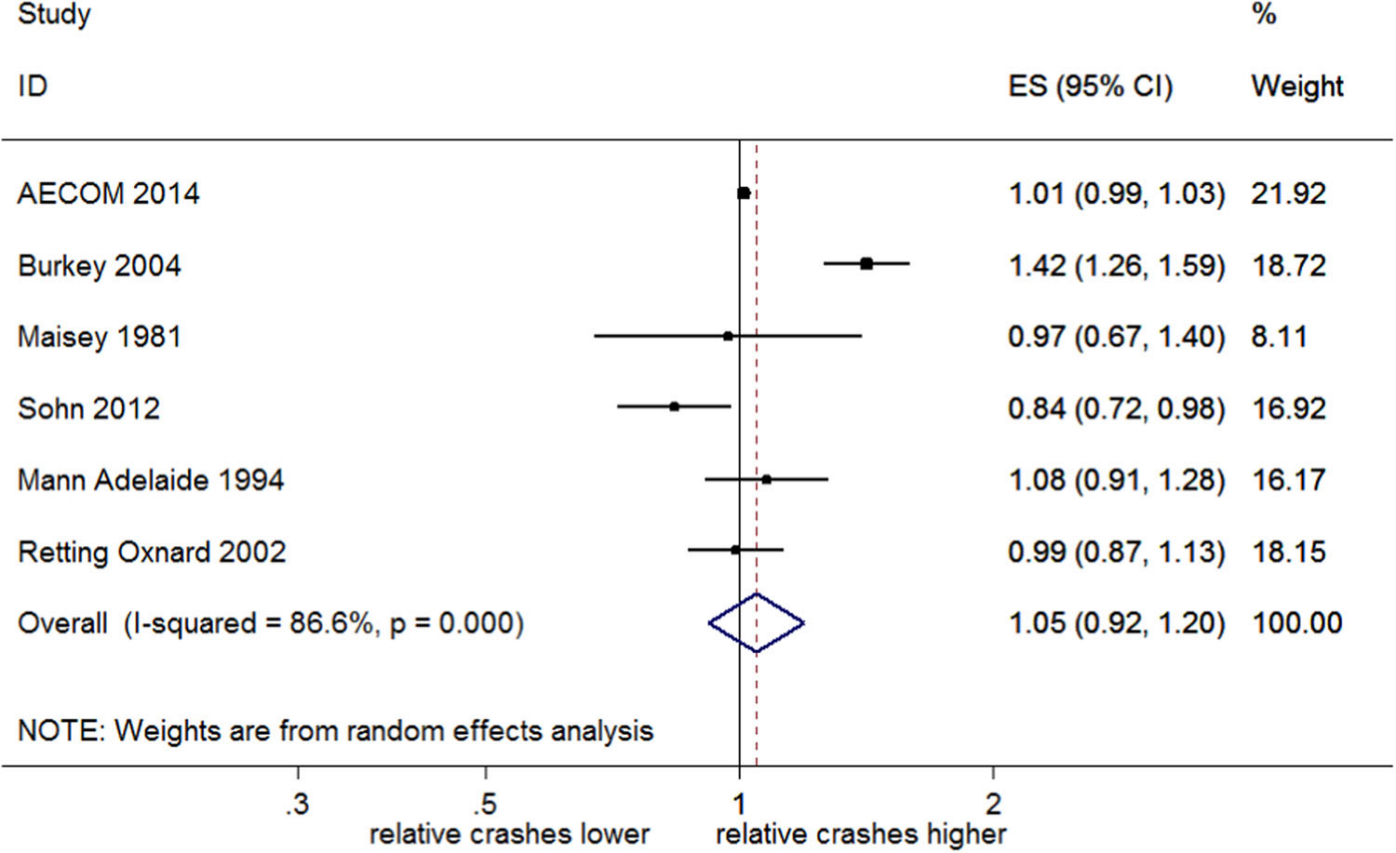
Effects of red light cameras on total property damage‐only crashes. CI, confidence interval; ES, effect size

Seven studies examined PDO crashes, six of which included CIs or *SE*s. These are shown in Figure 6. Overall, there was a nonsignificant increase of 5% in PDO crashes (95% CI; 8% decrease–20% increase). It was not possible to obtain *SE*s from one study (Sayed & de Leur, 2007), and this reported reductions in PDO crashes of 14.3% after RLC implementation.

##### Total crashes from RLR

Six studies looked specifically at RLR crashes (producing seven estimates of effect). RLR crashes were identified as those caused directly by a driver running a red light or failing to yield during a turn on red, or any crash where a red light violation ticket was issued. The pooled estimates showed a 47% overall reduction in the total number of RLR crashes, although this was nonsignificant (95% CI; 62% decrease–2% increase; *I* ^2^ = 98.4%; *p *< .001).

**Figure 7:**
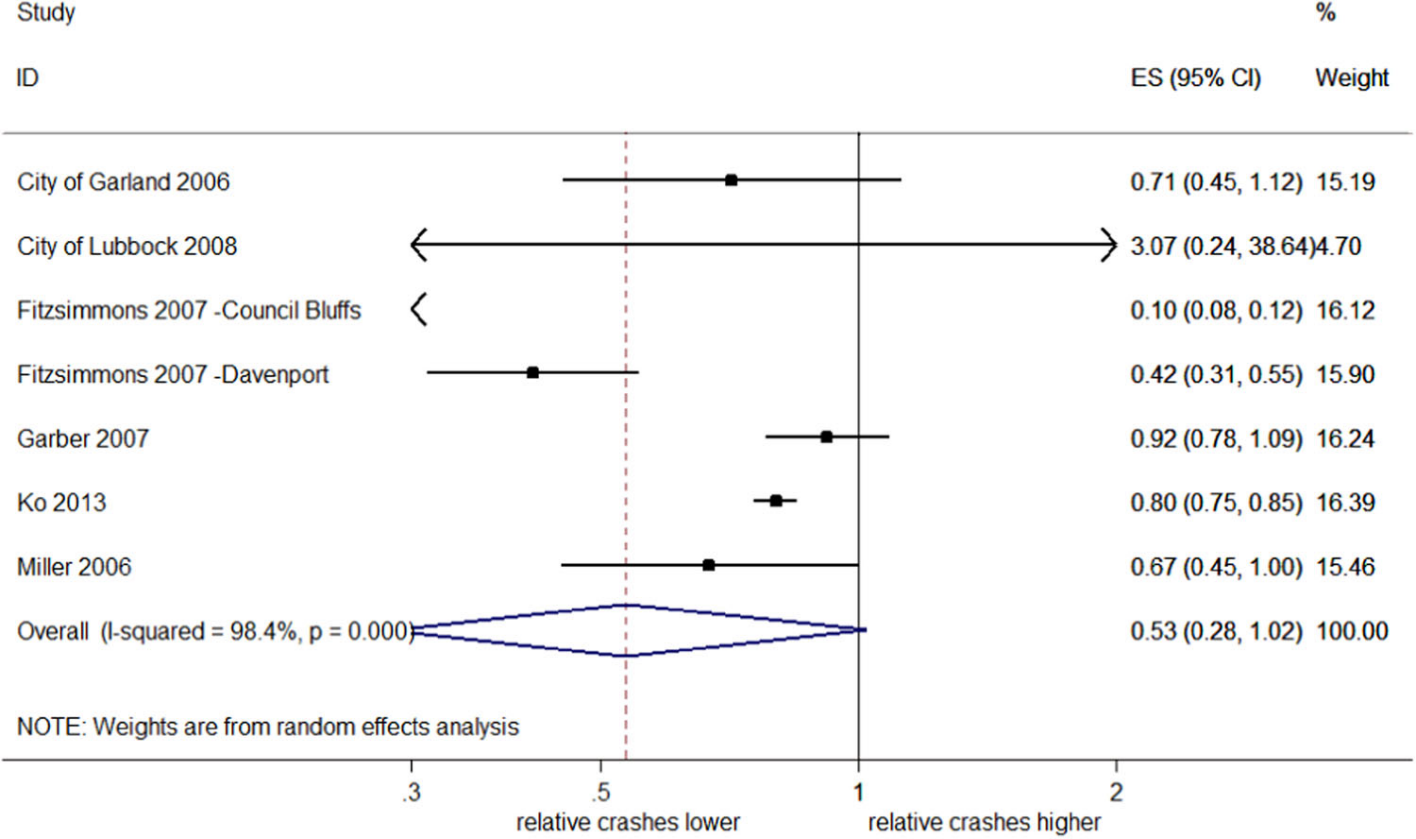
Effects of red light cameras on total crashes from red light running. CI, confidence interval; ES, effect size

Figure 7 identifies Fitzsimmons' Council Bluffs study (2007a, 2007b, 2007c) and Hallmark et al. (2010) as a visual outlier due to the extremely large reductions in RLR crashes found in this study. As a sensitivity analysis, the meta‐analysis was repeated excluding this study; the direction of the overall estimate of effect was unchanged but the estimated reduction in crashes dropped by over half to 18%, which was now significant (95% CI; 25–11% decrease). Removing this study also reduced heterogeneity (*I* ^2^ = 14.9%; *p *= .320). When City of Lubbock (2008) was also removed from the pooled analysis, RLR crashes were significantly reduced by 19% (95% CI; 25–11% decrease) and heterogeneity remained low (*I* ^2^ = 17.5%, *p *= .304).

Two further studies reported estimates of effect of RLCs on road traffic crashes from RLR. However, these limited “total” crashes to only specific types, rather than all crashes due to RLR. Therefore, they were not considered comparable and were excluded from the pooled analysis. Andreassen (1995) reported a 7% increase in total RLR crashes, which were defined as the sum of pedestrian, right angle, turning (same roadway) and rear end crashes. Cunningham and Hummer (2010) similarly reported a 5% increase in RLR crashes, which were defined as the sum of turning (both same roadway and different roadways), right angle and rear end crashes.

##### Total injury crashes from RLR

Two studies (not enough for a pooled analysis) specifically examined RLR crashes that resulted in injuries. Miller et al. (2006) reported a 34% reduction (95% CI; 64–5% decrease) while Garber (2007) reported a nonsignificant increase of 7% (95% CI; 18% decrease–31% increase).

#### 5.3.2 Right angle crashes

##### Total right angle crashes

**Figure 8:**
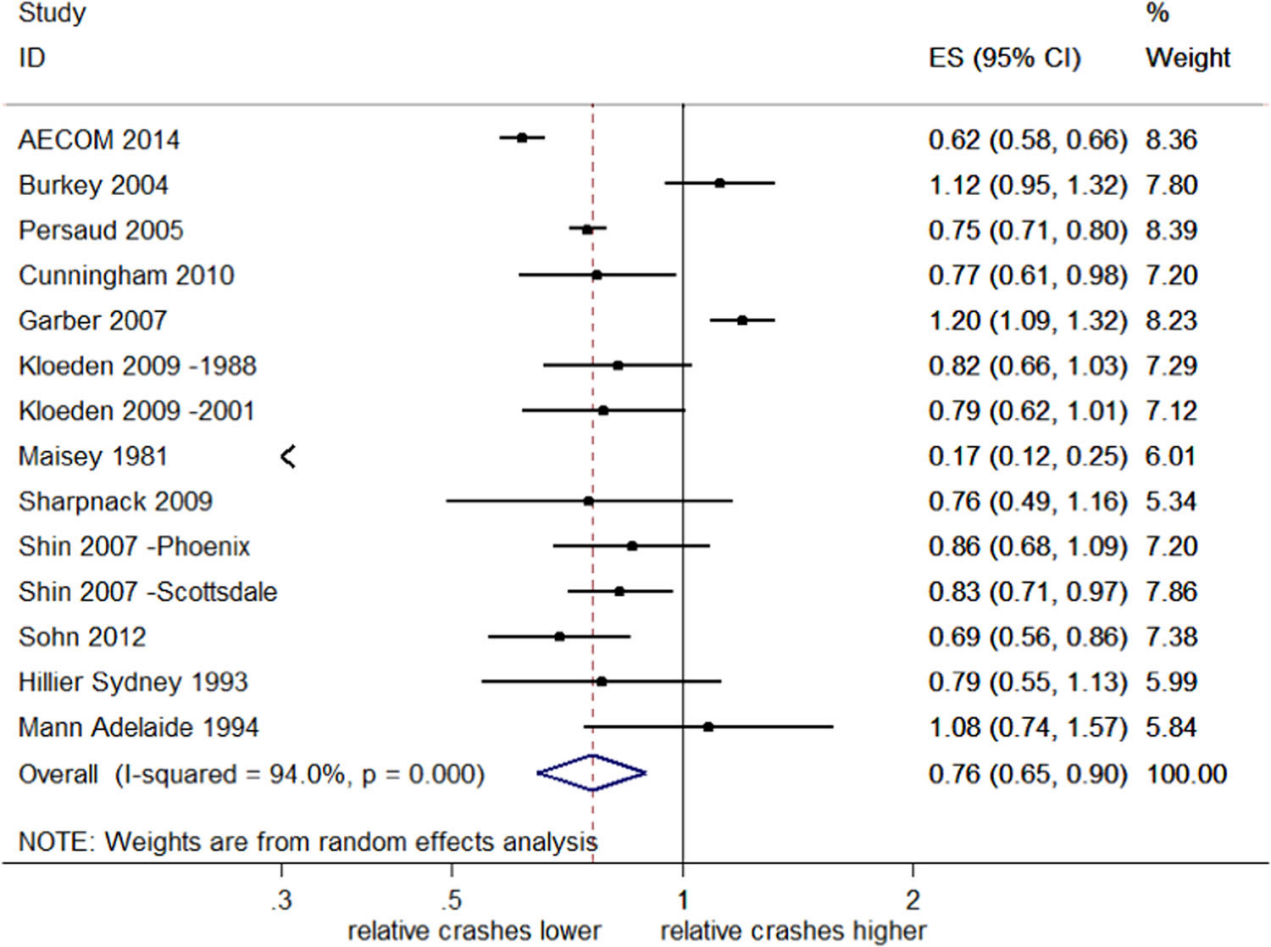
Effects of red light cameras on total right angle crashes. CI, confidence interval; ES, effect size

Fifteen studies evaluated the effect of RLCs on total right angle crashes (including property only crashes), providing 17 estimates of effect. Twelve of these studies (14 estimates of effect) included CIs. Garber (2007) and Persaud et al. (2005) provided both overall estimates of effect and estimates broken down by jurisdiction; the combined overall effects reported in each of these studies have been included in the pooled analysis as it was considered that the different geographic regions would not be independent. Figure 8 indicates a significant reduction in right angle crashes of 24% at intersections with RLCs compared with those without (95% CI; 45–10% decrease) with evidence of heterogeneity between studies (*I* ^2^ = 94%, *p *< .001).

Three newly identified studies reported results from which *SE*s were incalculable (Andreassen, 1995; Richardson, 2003; Sayed & de Leur, 2007). Andreassen (1995) reported mixed findings with some sites showing an initial decrease in crashes in years following RLC installation, and sites with fewer crashes reporting increased crashes in later years. Using an EB analysis, Richardson (2003) reported an increase of 0.07 crashes per site per year as a result of RLC installation which was equated to an increase of 6% in right angle crashes per year. Sayed and de Leur (2007) reported reductions in right angle crashes of 17.2% after RLC implementation.

##### Total right angle crashes stratified by country

**Figure 9:**
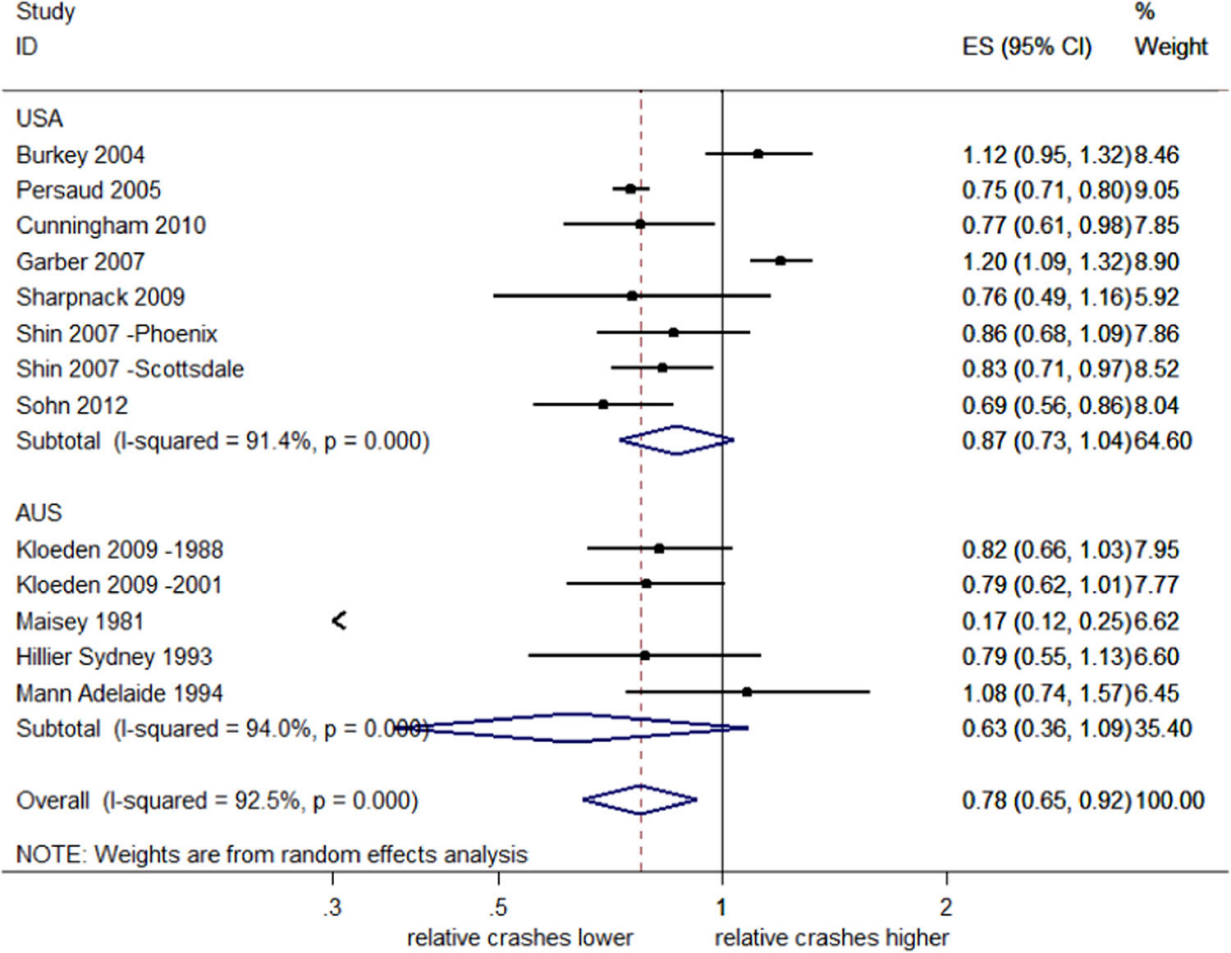
Effects of red light cameras on total right angle crashes—stratified by country. CI, confidence interval; ES, effect size

As shown in Figure 9, a subgroup analysis by country suggested a nonsignificant decrease of 13% in right angle crashes in the United States (95% CI; 27% decrease–4% increase) and 37% in Australia (95% CI; 64% decrease–9% increase). There was evidence of significant heterogeneity in both countries (*I* ^2^ = 91.4%, *p *< .001 in the United States and *I* ^2^ = 94%, *p *< .001 in Australia) and no evidence of a difference in effect between subgroups (*Q *= 1.21, *p *= .27).

##### Right angle injury crashes

**Figure 10:**
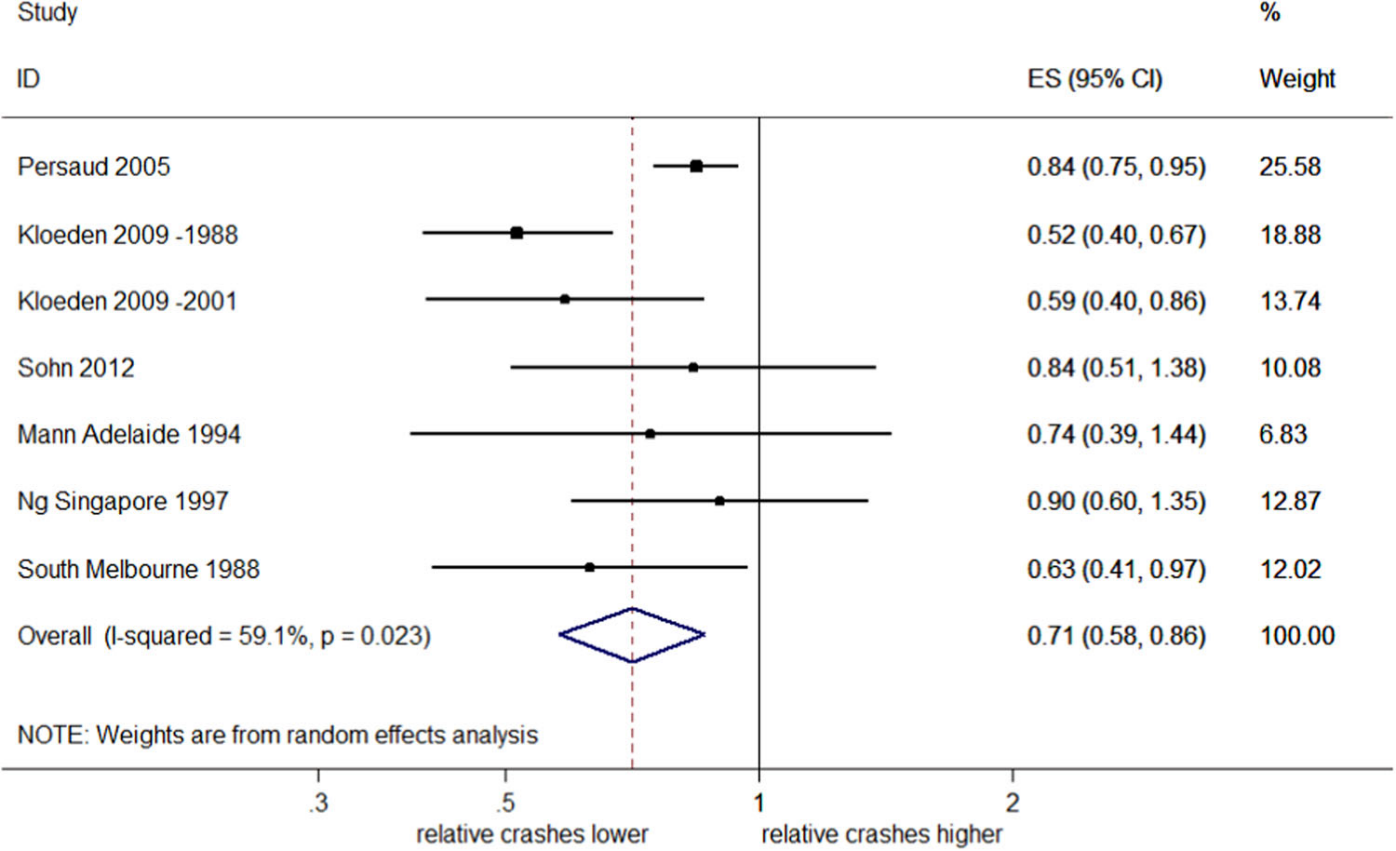
Effects of red light cameras on right angle injury crashes. CI, confidence interval; ES, effect size

Seven studies reported eight estimates of effect; one of these studies (Kull, 2014) reported effect estimates without CIs. The pooled analysis of studies with reported CIs, which is shown in Figure 10, estimates a significant overall reduction of 29% in right angle injury crashes (95% CI: 42–14% decrease), with moderate evidence of heterogeneity (*I* ^2^ = 59.1%, *p *= .023).

##### Right angle injury crashes stratified by country

**Figure 11:**
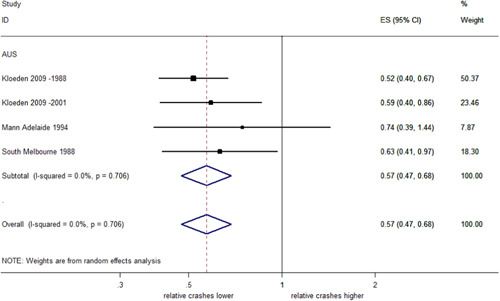
Effects of red light cameras on right angle injury crashes—stratified by country. CI, confidence interval; ES, effect size

There were not enough studies to estimate the pooled effect of RLCs on right angle crashes in the USA. In Australia, a pooled estimate indicated a significant 43% decrease (95% CI: 53–32% decrease) with no evidence of heterogeneity (*I* ^2^ = 0%, *p* = .706), as shown in Figure 11.

##### Right angle crashes from RLR

Only Ko et al. (2013) reported right angle crashes that resulted directly from RLR (including PDO crashes). This study estimated a significant reduction of 24% (95% CI: 28–19% decrease).

#### 5.3.3 Other turning crashes

A number of studies examined different crash types, such as turning, same roadway crashes (where cars approaching from opposite directions on the same road crash when one turns across the path of the other); turning, different roadway crashes; total direct and indirect right angle crashes; and the sum of all turning and right angle crashes at intersections. Most of these outcomes were unique to individual studies and could not be compared to other reported outcomes; however, a few studies reported turning, same roadway crashes. These have been pooled to give an overall estimate of effect.

##### Total turning, same roadway crashes

Five studies reported turning, same roadway crashes as an outcome with seven estimates of effect. Two of these studies reported effect estimates without CIs (Andreassen, 1995; Richardson, 2003).

**Figure 12:**
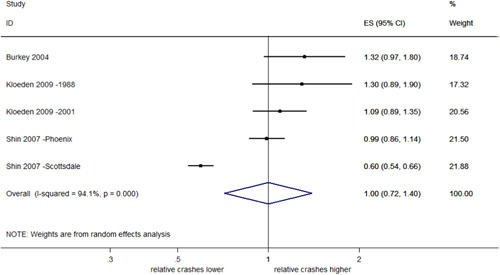
Effects of red light cameras on total turning, same roadway crashes. CI, confidence interval; ES, effect size

The pooled analysis, shown in Figure 12, estimates no effect of RLCs on turning, same roadway crashes (95% CI: 28% decrease–40% increase), with substantial heterogeneity (*I* ^2^ = 94.1%, *p *< .001). The two studies without CI/*SE* Using before and after crash rates, Andreassen (1995) found a marginal increase in turning same roadway crashes after RLC implementation, while Richardson (2003) reported a decrease of 0.04 crashes per site per year as a result of RLC installation which was equated to a decrease of 2.4% in turning same roadway crashes per year.

##### Turning, same roadway injury crashes

Only Kloeden et al. (2009) reported turning, same roadway crashes that resulted in injury with CIs (producing two estimates of effect). South et al. (1988) and Kull (2014) also reported estimates of effect for this outcome without CI/*SE*s. Kull (2014) reported a reduction in turning injury crashes of 0.33 crashes per intersection per year following RLC implementation while South et al. (1988) reported an increase of 2%.

#### 5.3.4 Rear end crashes

##### Total rear end crashes

**Figure 13:**
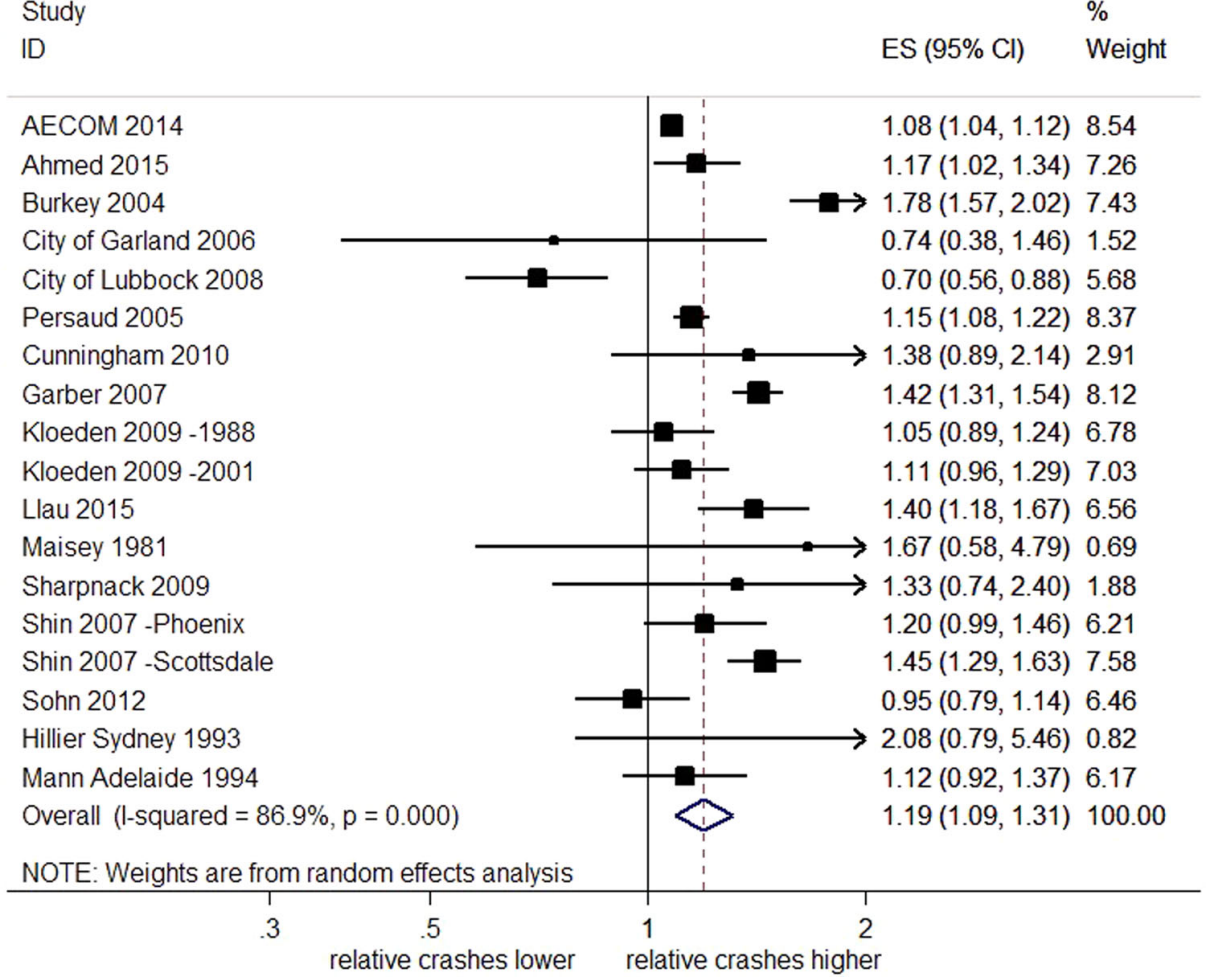
Effects of red light cameras on total rear end crashes. CI, confidence interval; ES, effect size

Nineteen studies evaluated the effect of RLCs on total rear end crashes, reporting 21 estimates of effect. Sixteen of these studies (18 estimates of effect) reported CI/*SE*. Overall, these 16 studies showed significant evidence for an increase of 19% in rear end crashes with RLCs (95% CI: 9–31% increase). This is shown in Figure 13.

Three studies were found where *SE*/CIs could not be obtained. Andreassen (1995) reported a 20% increase at RLC sites in comparison to control intersections. Using an EB analysis, Richardson (2003) reported an increase of 0.16 rear end crashes per site per year as a result of the installation of RLCs, this equated to a 9.2% increase per year. Sayed and de Leur (2007) reported a reduction in rear end crashes after RLC implementation of 12.4%.

##### Total rear end crashes stratified by country

**Figure 14:**
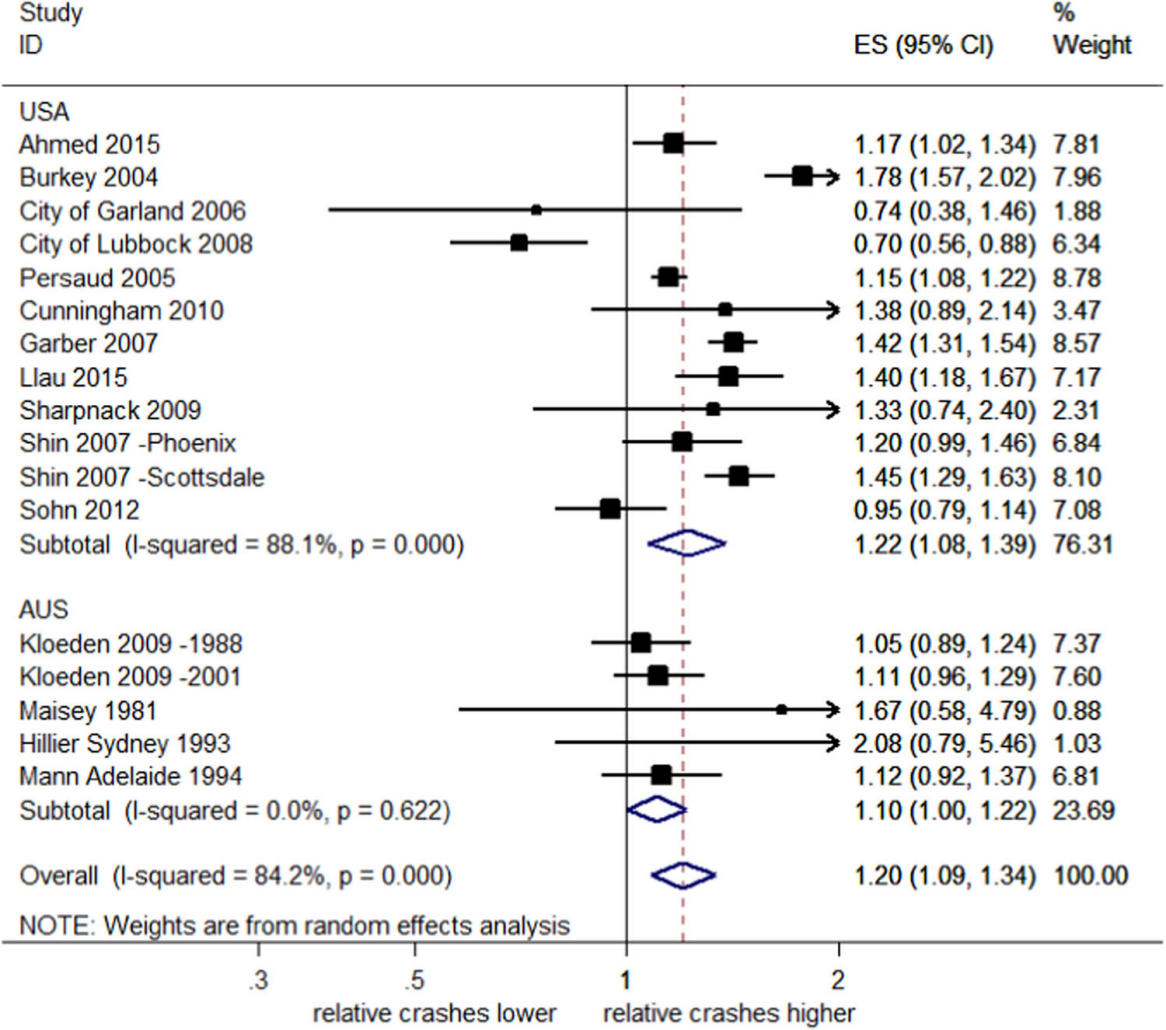
Effects of red light cameras on total rear end crashes—stratified by country. CI, confidence interval; ES, effect size

A meta‐analysis of studies in the United States found a significant 22% increase in rear end crashes (95% CI: 8–39% increase), with evidence of heterogeneity (*I* ^2^ = 88.1%, *p *< .001). Pooled estimates from Australia indicated a significant increase of 10% (95% CI: 0–22% increase), with no evidence of heterogeneity (*I* ^2^ = 0%, *p *= .622). This is shown in Figure 14. The test of group differences indicates no evidence against the null hypothesis that the subgroup effects are the same (*Q *= 1.52, *p *= .22).

##### Total rear end injury crashes

**Figure 15:**
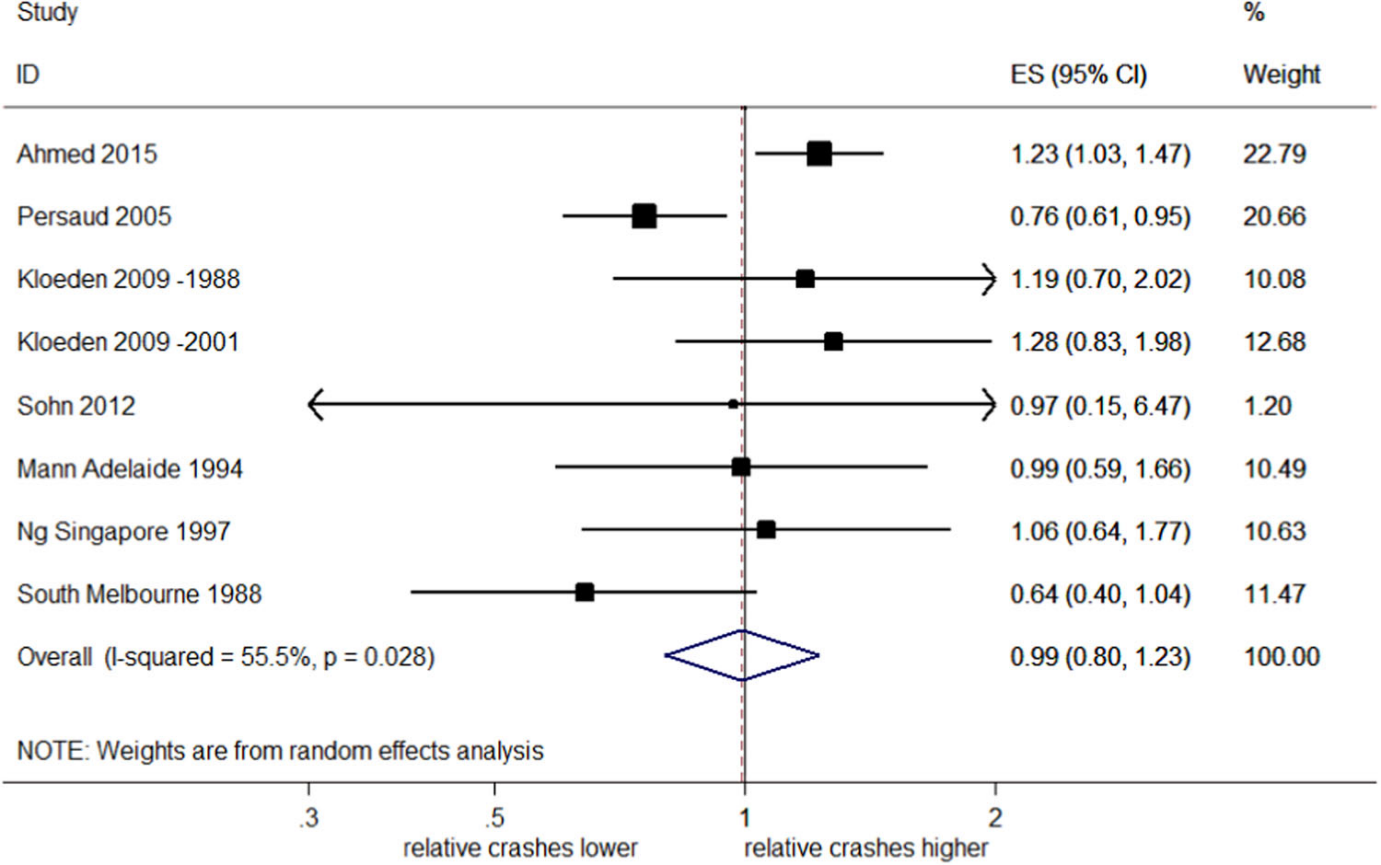
Effects of red light cameras on total rear end injury crashes. CI, confidence interval; ES, effect size

Eight studies (nine estimates of effect) examined total rear end crashes that resulted in injury; of these seven studies reported eight estimates of effect with CI/*SE*. The pooled analyses of these eight estimates, which is shown in Figure 15, suggested a nonsignificant decrease in rear end injury crashes of 1% (95% CI: 20% decrease–24% increase) and moderate heterogeneity (*I* ^2^ = 55.5%, *p *= .028).

Using annual rear end injury crash rates, Kull (2014) found an increase of 17% following RLC installation.

##### Rear end injury crashes stratified by country

**Figure 16:**
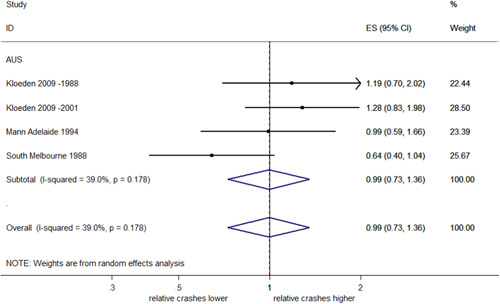
Effects of red light cameras on total rear end injury crashes—stratified by country. CI, confidence interval; ES, effect size

There were not enough studies from the United States to estimate a pooled effect on rear end injury crashes. In Australia, a meta‐analysis found a nonsignificant 1% reduction in rear end injury crashes (95% CI: 27% decrease–36% increase) with no evidence of heterogeneity (*I* ^2^ = 39%, *p* = .178). This is shown in Figure 16.

##### Total rear end crashes from RLR

**Figure 17:**
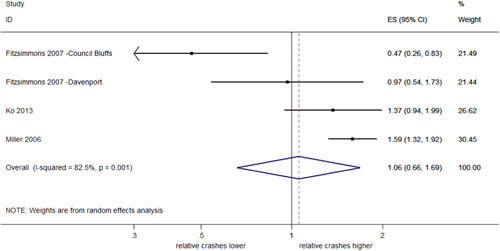
Effects of red light cameras on total rear end crashes from red light running. CI, confidence interval; ES, effect size

Three studies estimated the effect of RLCs on total rear end crashes that were specifically identified as resulting from a red light violation (four estimates of effect). As seen in Figure 17, a pooled estimate indicated a nonsignificant 6% increase in rear end crashes from RLR (95% CI: 34% decrease–69% increase) with evidence of heterogeneity (*I* ^2^ = 82.5%, *p *< .001).

#### 5.3.5 Red light violations

Three studies estimated the effect of RLCs on the number of red light violations, although only two of these (Arup Transportation Planning, 1992; Retting et al., 1999a) reported CI/*SE*s. Arup Transportation Planning (1992) reported a significant decrease of 61% (95% CI: 50–70% decrease), while Retting et al. (1999a) reported a nonsignificant decrease of 47% (95% CI: 83% decrease–66% increase). One additional study (Chin, 1989) where CIs could not be obtained, reported rates of red light violation per red light cycle and found a reduction of around 44% after RLC installation.

#### 5.3.6 Additional subgroup analyses

Additional analyses were conducted with studies stratified based on whether studies accounted for RTM, whether or not RLC programs mentioned the use of warning signs, and whether or not the studies were peer‐reviewed prior to publication. These subgroup analyses did not consistently explain the heterogeneity between‐study results (forest plots not shown). However, studies accounting for regression to the mean tended to report more moderate decreases for right angle injury crashes than studies not accounting for regression to the mean. Similarly, peer‐reviewed studies reported more moderate decreases for right angle crashes resulting in injury than studies that were not peer reviewed prior to publication (forest plots in Appendix H). Subgroup analyses were also conducted with studies stratified according to risk of bias across the six domains. These analyses did not invariably explain the heterogeneity between‐study results. However, studies that had a low risk of bias in terms of controlling for confounders were found to report a greater increase in rear end crashes than studies with a high risk of bias. Studies with low risk of bias due to control of confounding reported a nonsignificant decrease in right angle crashes, compared with studies with a high risk of bias that reported a significant decrease. When stratified by risk of bias according to control for confounders and other potential sources of bias, there was some evidence that the subgroup effects on total crashes differed. The effect was inconsistent and reflected the highly heterogeneous results otherwise found for total crashes (forest plots of additional subgroup analyses in Appendix H).

#### 5.3.7 Summary of effects

This systematic review shows that RLCs resulted in a significant reduction of of 24% in right angle crashes (95% CI: 35%–10% decrease), of 29% in right angle injury crashes (95% CI: 42%–14% decrease), and of 20% in total injury crashes (95% CI: 32%–5% decrease; see Table 3). A large reduction in red light violations was suggested; however, only two of three studies reported CIs preventing appropriate meta‐analysis. RLCs are also associated with a significant increase in rear end crashes of 19% (95% CI: 9%–31% increase).

**Table 3:** Summary of overall effect estimates

Outcome		Overall ES	95% CI	I2%	*p*‐Value	No. studies (no. estimates)
Total crashes	↓	0.98	0.91–1.07	90.4	.000	20 (23)
Total injury crashes	**↓**	0.80	0.68–0.95	93.1	.000	15 (16)
PDO crashes	↑	1.05	0.92–1.20	86.6	.000	6 (6)
Total RLR crashes	↓	0.53	0.28–1.02	98.4	.000	6 (7)
Right angle crashes	**↓**	0.76	0.65–0.90	94.0	.000	12 (14)
Right angle injury crashes	**↓**	0.71	0.58–0.86	59.1	.023	6 (7)
Rear end crashes	**↑**	1.19	1.09–1.31	85.9	.000	16 (18)
Rear end injury crashes	↓	0.99	0.80–1.23	55.5	.028	7 (8)
Rear end RLR crashes	↑	1.06	0.66–1.69	82.5	.000	3 (4)

*Note*: Arrows denote direction of effect, with bold indicating a significant result. Only results for meta‐analyses where all studies included *SE*s are included.

Abbreviations: CI, confidence interval; ES, effect size; PDO, property damage only; RLR, red light running; *SE*, standard error.

There was no evidence to suggest that study heterogeneity was consistently explained by country, although results were more consistent across outcomes for studies conducted in Australia. Additional subgroup analyses found some evidence that when stratified by risk of bias according to control for confounding, studies with a low risk of bias reported a nonsignificant reduction in right angle crashes.

### 5.4 Mechanisms

Mechanisms focus on how and why an intervention works. There are clearly a number of reasons why drivers may intentionally run red lights, including being in a rush and needing to save time, impatience, and frustration with having to stop (see e.g., Wissinger, Hummer, & Milazzo, 2000; Porter & Berry 1999). A review of the relevant research suggests that the primary mechanism by which RLCs may reduce RLR is deterrence, which involves the use or the threat of punishment to discourage people from offending. A detailed discussion of deterrence theory, as well as a review of the issue of spillover and the halo effect may be found in the Discussion.

### 5.5 Moderators

Moderators are “variables that may explain outcomes across different studies” (Johnson et al., 2015, p.462). Possible moderators could include factors such as road type, intersection geometry, traffic flow, number of lanes, speed limits, weather conditions, and country or area level effects. In the meta‐analysis, studies were stratified by country (United States and Australia) to examine country‐level effects, with further subgroup analyses conducted on risk of bias, RTM, and the use of warning signs. However, there was insufficient data to allow further analysis of other potential moderators in this manner. Some of the individual studies did note, discuss, and in some cases control for a number of possible moderating factors. However, there was only limited evidence regarding their effect on RLC program outcomes.

#### 5.5.1 Time of day and day of week

Time of day and day of week have been suggested as possible moderators for RLC effects. Retting, Williams, and Greene (1998); Porter and Berry (1999); and Kamyab, McDonald, and Stribiak (2000) all found that the incidence of red light violations is greater on weekdays than weekends. They also found that red light violations varied by time of day: Retting et al. (1998) and Porter and Berry (1999) indicated that the average number of red light runners was greater during the day than at night while Kamyab et al. (2000) reported a higher incidence of red light violations specifically between 3:00 pm and 5:00 pm. Fitzsimmons (2007a) suggested that these may be related to traffic volumes and congestion levels, pointing out that drivers who are on the road during peak hours may experience delays, which may lead to adverse behavior such as red light violations, particularly if traffic signals are improperly timed. Conversely, Arup Transportation Planning (1992) found that prior to the installation of RLCs, the number of violations per hour was lower during peak periods (defined as 7:30 am to 9:30 am and 4:00 pm to 6:00 pm), regardless of traffic flows. However, they also stated that during these peak periods, signal coordination was designed to favor major traffic flows, reducing delays and decreasing the potential for violations.

It should be noted that some of the studies in the meta‐analysis did not collect data around the clock. Chin (1989), for example, only collected data on weekdays and only during specific periods of the day that fell between 8:00 am and 7:00 pm.

#### 5.5.2 Signal timing

Another possible moderator is signal timing. Retting and Greene (1995) found that longer change intervals, which may be created by increasing the length of the yellow signal interval, may reduce red light violations and traffic crashes. Bonneson and Son (2003) reported that RLR rates are higher when the duration of the yellow signal interval is shorter than suggested by traffic engineering equations and Bonneson and Zimmerman (2004) found that after increasing the yellow interval duration, there was a decrease in the frequency of red light‐related crashes. Retting, Ferguson, and Farmer (2008) found that longer yellow signal lengths were associated with a 36% decrease in red light violations.

#### 5.5.3 Speed limit variations

Variations in the speed limit on the roads used in RLC studies may also serve as a moderator variable. Some studies in the meta‐analysis did control for this, such as Miller et al. (2006) and Fitzsimmons (2007a, 2007b, 2007c) and Hallmark et al. (2010) considered the posted speed limit when selecting control intersections that were similar to the locations where RLCs were posted. Miller et al. (2006) only used roads with speed limits ranging from 35 to 55 mph; they pointed out that the effects of RLCs on roads with different characteristics, including speed limits, could vary.

#### 5.5.4 Other potential moderators

Other factors that could influence the results include average daily traffic, the presence and/or number of left turn lanes (equivalent to a right turn lane in the United Kingdom), the size or openness of the intersections (typically larger in the United States and Australia in comparison to the United Kingdom), road gradient, and the percentage of heavy trucks in the traffic stream. Additionally, whether the signals at the intersection were actuated, pretimed, or coordinated may affect driver behavior. An actuated traffic signal will change its phase if a vehicle is detected, while pretimed signals are preprogrammed to maintain constant signal intervals regardless of traffic flow. Coordinated signals are found along major roads with multiple traffic signals and are timed to allow drivers traveling the road at the posted speed limit to have all green signal phases.

A UK study conducted on RLR in Birmingham (Lawson, 1991) reported some “average” characteristics associated with increased RLR; these features may also moderate the effect of RLCs across different studies. They found RLR crashes were relatively common late at night and in the early hours compared with non‐RLR crashes, particularly on Saturday night and Sunday morning. The study reported that a combination of high speeds, large traffic volumes, and a sense of openness at the intersection could all contribute to increased RLR crashes. A sense of openness could be created by a combination of a downhill gradient toward an intersection, clear visibility through a stretch of road on the intersection exit, or the presence of multiple lanes. Lawson also noted a great variation in crash patterns between intersections and within approaches of intersections, and suggested crash history at individual sites, rather than general intersection characteristics, is the best indicator of where to place RLCs.

### 5.6 Implementation

Implementation involves the context in which RLC programs are put into practice. Variations in implementation may influence program effectiveness, affecting the level of deterrence. The structure and organization of RLC programs vary greatly by jurisdiction and a number of these variations may affect how driver behavior is influenced by RLCs, although most were not tested directly by the included studies.

#### 5.6.1 Public awareness and program publicity

Public knowledge of the implementation of a photo enforcement program is considered essential for program success. The use of publicity campaigns to enhance public awareness, as well as posted warning signs (discussed below), are believed to increase the general deterrent effect of the cameras and to create spillover from intersections with cameras to the wider area (see e.g., Ross & Sperley, 2011; Shin & Washington, 2007). Retting and Kyrychenko (2002) stated that the primary mechanism for preventing red light violations is driver awareness and stressed the importance of publicizing RLC enforcement programs.

In the United States, the Federal Highway Administration (FHA, 2004b) stated that a critical element for proper implementation of RLCs is an ongoing public education program. Similarly, in the United Kingdom, the Department for Transport (2007, p.9) stated, “Every effort should be made to publicize the use of cameras in an area.” The Department suggests that such publicity will enhance the deterrent effect of camera programs and improve public compliance with traffic laws.

Many of the included studies specifically mentioned the use of public awareness programs and media publicity regarding the operation of RLCs. Some jurisdictions mailed written notices to local residents to alert them to the new photo enforcement program. In many cases, the programs began publicity campaigns months before the cameras were installed and some even provided specific information as to which intersections were selected for RLC monitoring. To further increase awareness, and possibly enhance specific deterrence, many jurisdictions implemented a warning period prior to beginning formal RLC enforcement. During this period, violators were issued warnings rather than being ticketed. Warning periods, when used, generally lasted 30 days or less (see, e.g., Fitzsimmons, 2007a, 2007b, 2007c; Hallmark et al., 2010; Llau et al., 2015; Porter, Johnson, & Bland, 2013; Retting & Kyrychenko, 2002; Retting et al., 1999a; Ross & Sperley, 2011).

While none of the included studies specifically examined the impact of publicity programs on camera effectiveness, many emphasized the importance of these programs. As Ross and Sperley (2011, p.9) pointed out, “To maximize the impact of red light camera enforcement, drivers must be aware of the enforcement…”

#### 5.6.2 Warning signs

A number of the included studies reported that the jurisdictions under study posted signs to inform drivers that RLCs were in use (see Figure 19 for a complete list). Along with publicity campaigns, warning signs are designed to increase driver awareness of the automated enforcement programs and to enhance their deterrent effect.

Warning signs may be posted either at or near the specific intersections at which cameras are installed or at major entrance points to the city (or both). In some cases, the use and placement of signage may be affected by legislative requirements; in the United States, this varies by state. In North Carolina, for example, state statutes require warning signs to be posted on all approaches of any intersection at which an RLC is installed, regardless of which approaches are actually monitored by RLCs (Cunningham & Hummer, 2010). Conversely, in Oregon, cities operating RLCs are required to post signs on all major routes into the city; Ross and Sperley (2011) reported that Salem, Oregon also posted warning signs on each approach where a camera was operating to help increase driver awareness of photo enforcement. In California, warning signs are required but local governments are given the option of placing signs either at each monitored intersection or at all major city or county entrances (see e.g., California State Auditor, 2002; Retting & Kyrychenko, 2002; Retting et al., 1999a). However, using the latter option does mean that there is a risk that some major entrances may be overlooked. California State Auditor (2002) reported that in 1999 approximately 700 citations for red light violations were dismissed in Sacramento after a traffic court ruling found the city had failed to fully comply with the law when installing warning signs. Following this ruling, the city placed warning signs at each RLC‐monitored intersection.

The location of warning signs may affect their utility. California State Auditor (2002) suggested that placing warning signs at major entrance points rather than at treated intersections would enhance the deterrent effect of RLCs across the entire jurisdiction. Kloeden et al. (2009) reported that in Adelaide, Australia an informal survey found that many people were unaware of the location of RLCs and suggested that the location of the signs, which in Adelaide were frequently placed well back from the intersection being monitored and off to the side of the road, may have contributed to the lack of knowledge about the program and to the lack of effectiveness of RLCs in reducing traffic crashes in 2001. They suggested that placing warning signs on the far side signal poles might improve the effects of cameras.

Only a small number of studies examined the impact of signage on RLC effectiveness. Shin and Washington (2007; as discussed in Washington, 2005) stated that angle and left‐turn crash reduction benefits from RLCs appeared to be greater at intersections where warning signs were installed than at intersections without signage. Persaud et al. (2005; as discussed in Council, Persaud, Eccles, Lyon, & Griffith, 2005) reported that the use of warning signs at intersections was associated with a smaller benefit than warning signs at both intersections and city limits.

**Figure 18:**
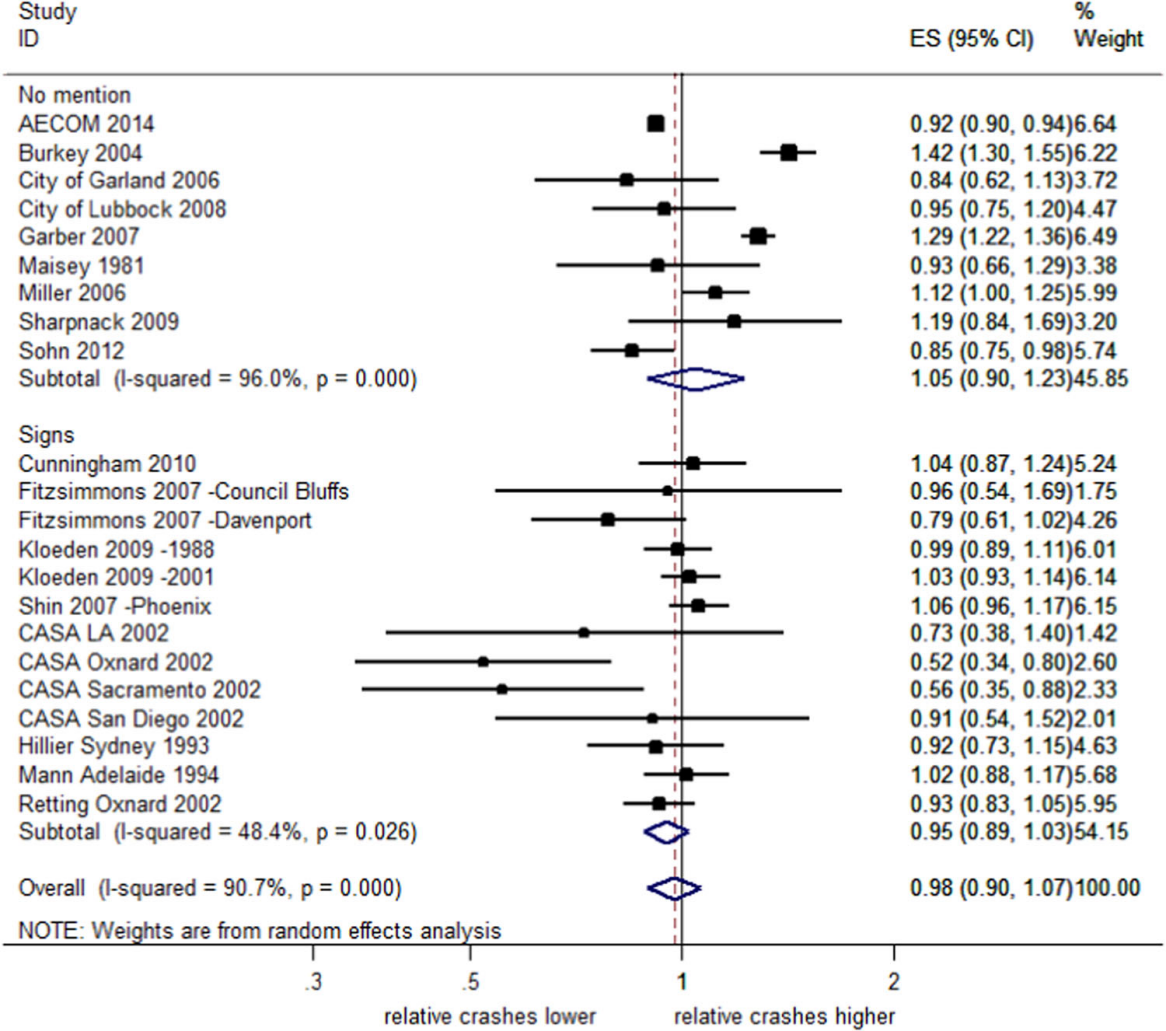
Effects of red light cameras on total crashes—stratified by the use of warning signs. CI, confidence interval; ES, effect size

**Figure 19:**
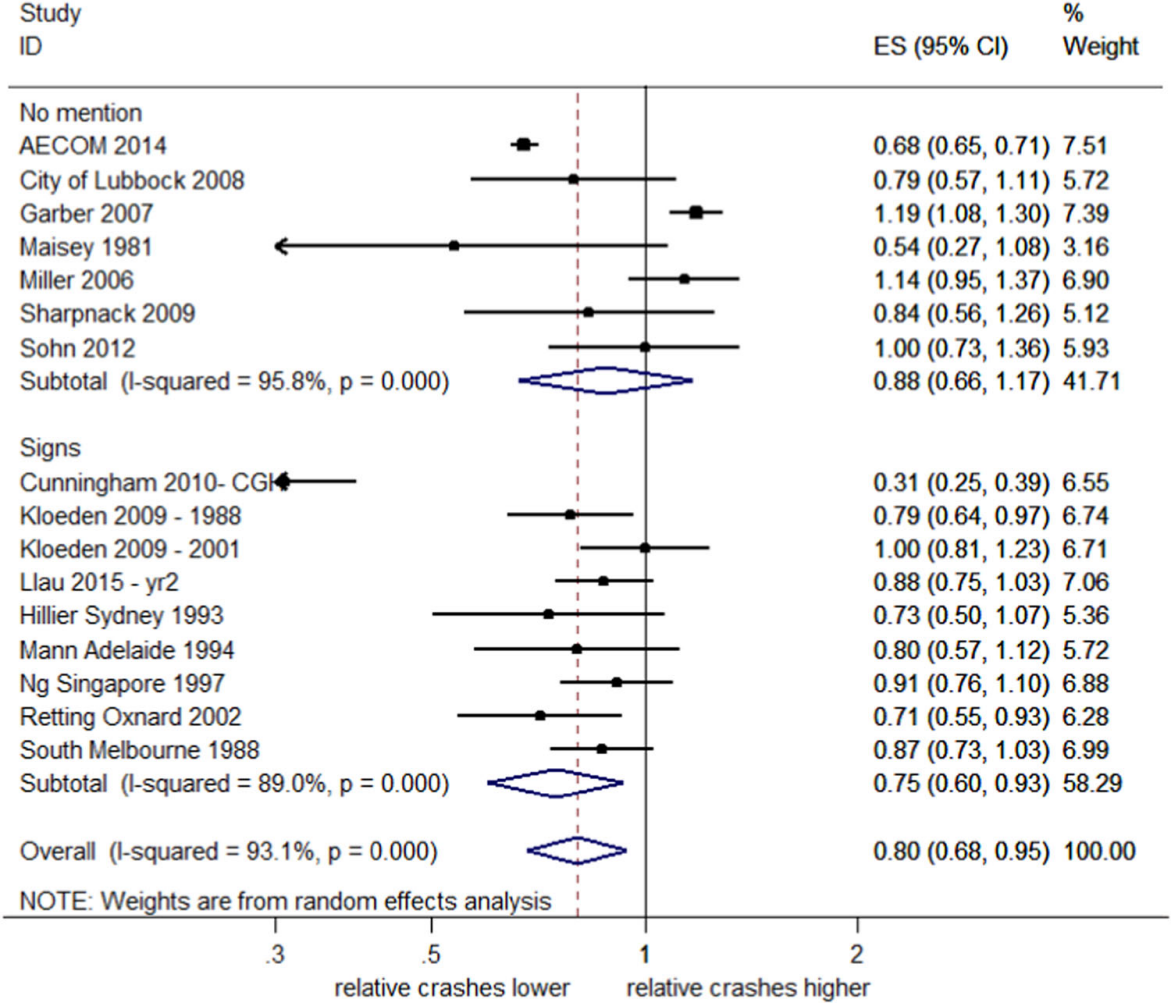
Effects of red light cameras on total injury crashes—stratified by the use of warning signs. CI, confidence interval; ES, effect size

A meta‐analysis was conducted to explore the effect of warning signs on total crashes (Figure 18) and total injury crashes (Figure 19) in the primary studies. There was no significant difference in the effect of RLCs on total road traffic crashes or total injury crashes between the studies that mentioned the use of warning signs and those that did not. However, for both crash types, studies reporting the use of warning signs do show a slightly greater trend toward a reduction in crashes than studies that did not mention the use of warning signs (albeit insignificant).

#### 5.6.3 Obstacles to implementation

Very few of the included studies provided any detailed discussion of the types of implementation issues and obstacles that RLC programs face. However, several key obstacles that were mentioned included problems with contract vendors, issues involved in operating a legally‐compliant RLC program, and public attitudes and concerns.

##### Vendor‐related concerns

A vendor is the agency that supplies an RLC system for a community, and also operates the system in jurisdictions where the police do not do so. Sharpnack (2009) reported that numerous issues with the vendor threatened the success of the RLC program in Costa Mesa, California. Ongoing issues requiring multiple contacts every week with the vendor included inaccurate record keeping, billing errors, technology malfunctions, problems with traffic signal phasing, and performance issues. One serious issue was the inability of the vender to ensure that the cameras were obtaining clear images of drivers at RLC intersections; this problem led to a large number of citations being dismissed. Similar issues were reported by other studies (California State Auditor, 2002; Fitzsimmons, 2007a, 2007b, 2007c; Hallmark et al., 2010).

##### Public concerns about privacy

A number of studies have cited public concerns regarding the possible invasion of personal privacy as a significant obstacle to RLC compliance (California State Auditor, 2002; Garber, 2007, as discussed in Garber et al., 2005; Hobeika & Yaungyai, 2006; Ross & Sperley, 2011). RLCs collect an enormous amount of personal information about the vehicle and the driver, including the precise location of the vehicle at one or more specific dates and times. This information could be used to track an individual's location, with serious potential legal or even personal repercussions.

While police officers engaged in traditional traffic enforcement would gather the same information, in that situation the data would be controlled and protected by the police department. In many jurisdictions, RLCs are operated by for‐profit third‐party vendors rather than by the police, creating serious public privacy concerns. The ACLU (2011) stressed “the need for strict controls on what data is collected, how long it is held and by whom, and access to the data by third parties.” The concern is that the data may become accessible not only to traffic enforcement agencies but also to other governmental agencies and officers and to non‐governmental entities and may even become public record. This could result in a serious violation of personal privacy.

Another concern is possible misuse of the photographs taken by the RLCs. While California law prohibits the use of RLC photographs for any purpose other than the prosecution of the motorist for the traffic violation, California State Auditor (2002) reported that nearly all of the local California governments studied had or would use these photos for other purposes as well, including the investigation of unrelated criminal activities and as evidence in court.

##### Legal challenges

A number of studies reported issues relating to the legalities involved in operating an RLC program, particularly relating to issuing citations. California State Auditor (2002) reported that several local governments in California, including San Diego, Beverly Hills, and San Francisco, have been sued by red light violators who challenged program operations.

In the United States, state statutes generally require traffic citations be issued by law enforcement officers. If tickets resulting from RLC enforcement are sent out by a third party, such as the vendor operating the system, this may be a violation of the law. For example, a Florida state statute mandates that only law enforcement officers may issue violations. In 2015, traffic judges in Broward County, Florida, dismissed 24,000 pending RLC tickets, with a value of over $6.3 million. The judges stated that because the videos from the cameras were sent out of state for screening by an Arizona‐based vendor, the programme violated Florida law (Ballou, 2015). Similar challenges in other cities and counties around the state have led many jurisdictions to shut down their RLC programmes (Sweeney, 2017). Florida legislators have repeatedly attempted to outlaw RLCs in the state and in January 2018, the Florida House voted to repeal the law permitting the use of RLCs. However, as in the previous year, the bill did not pass in the Senate (Koh, 2018). Later the same year, the Florida Supreme Court reversed the finding of the Broward County court, unanimously ruling that private vendors are allowed to review RLC footage, although they may not make the final probable cause determination as to whether a traffic infraction was committed or whether the driver is to be cited (*Jimenez v. State of Florida*; SC16‐1976). A similar debate is currently ongoing in Texas; the governor wants to ban RLCs and a case regarding the legality of RLC tickets will soon be heard by the Texas Supreme Court, while at the same time, local governments such as the Dallas City Council support the use of RLCs (Kalthoff, 2018).

A number of due process concerns have also been raised. ACLU (2011) pointed out that citations generated by RLC enforcement hold the owner or lessee of the motor vehicle liable for the alleged violation regardless of who was actually driving the vehicle at the time, stating, “Guilt is presumed over innocence.” Additionally, because of the delay between the actual violation and the receipt of the ticket, which is sent by mail, the owner of the vehicle may have difficulty remembering the event, or even who was actually driving the vehicle on that day. This may make it extremely difficult, if not impossible, to make any reasonable defense to the citation.

In Chicago, a class‐action lawsuit recently was brought against the city alleging that the city violated due process in the method used to notify drivers of violations and of late fees for failure to pay fines on time. This is part of a larger scandal in the city alleging corruption and mismanagement of the program, including bribery, inconsistent enforcement, and the use of unfair criteria to issue tickets (Kidwell, 2016).

##### Public concerns about safety versus revenue

Another issue that has been raised is that of the purpose of RLC programs and the belief that generating revenue for the local government may take priority over improved public safety (see, e.g., Hobeika & Yaungyai, 2006). Simons (2016) states that while they may be intended as safety programs, “increasingly, the public sees them as money‐making scams that can actually make roads less safe.” Essentially, many view RLCs as “cash cows” and money machines. Cunningham and Hummer, (2010; as discussed in Cunningham & Hummer, 2004) argues that this is a legitimate concern because if conflicts arise between revenue and safety, a system that is revenue‐focused will not be able to maximize safety.

Because the vendors are for‐profit businesses, they have an incentive to increase ticket revenue, regardless of public safety implications. Similarly, local and state governments may rely on the revenue generated by these programs. In 2016, a California state senator proposed a bill to reduce the base fine for rolling turn violations in the state. The bill resulted from growing concerns that after installing RLCs in several California cities, these violations, which cause only 0.5% of all traffic crashes, were accounting for nearly all red light citations. These tickets generated huge sums for the cities; the amount of money cleared by Rancho Cordova from red light infractions increased by over 100% between 2012 and 2016 (from approximately $73,000 to $742,000) because of the installation of four RLCs. The high fine and associated fees and other penalties, which were primarily intended to apply to much more dangerous violations, have caused severe hardship to lower‐income drivers and the bill was not only approved by the state Senate but was supported by the state ACLU, the National Motorists Association, and the California Association of Highway Patrolmen. However, the bill died in the Assembly appropriations committee “after it was determined that it would cause a significant loss of revenue at the state and local levels” (Flynn, 2016). Programs that appear to be revenue‐driven rather than a mechanism to improve road safety may generate public opposition, making implementation and continued operation more difficult.

#### 5.6.4 Other implementation factors

Both the US Department of Transportation and the UK Department for Transport provide guidelines that address the implementation of traffic enforcement camera programs. The Department for Transport (2007) published a report on best practices for implementing RLCs (as well as speed cameras and combined red light and speed cameras, known as traffic safety cameras) in the United Kingdom; the report includes guidance on deployment, visibility, and signage. It is based in part on an influential study (Hooke et al., 1996) conducted in Birmingham. Similarly, the FHA (2005) produced a set of operational guidelines for implementing RLC systems.

Some of the key issues that should be considered prior to implementing an RLC program include:

- Location/intersection selection: The Department of Transport (2007) recommends reviewing intersection crash history over the preceding three to five years and deploying RLCs at intersections with an accident history that includes at least one accident involving a fatality or serious injury in the past 36 months. The FHA (2005) also recommends identifying intersections with high crash rates but also suggest identifying intersections that the police identify as having red light violation problems as well as those which have high rates of public complaints.
- Signage: Appropriate signage must be used when deploying RLCs. As noted above, in the United States, the placement of warning signs may be mandated by state legislation, but both the US and UK guidelines recommend warning signs be posted even if they are not required by law or ordinance. Signs should be visible at all times and should not be obscured in any way; the FHA (2005) recommends regularly monitoring signs to ensure they are cleared of any vegetation growth that may interfere with visibility.
- Publicity: Both the US and UK guidelines emphasize the importance of publicizing the RLC program. The campaign should not only include information about the program itself but also educate the public about the dangers of RLR and emphasize the RLC program's goal of enhancing road safety. Publicity needs to be ongoing to counteract habituation and to maintain drivers' awareness of the risk of detection.
- System Assessment/Monitoring: Ongoing monitoring of RLC enforcement program efforts is essential to measure the effectiveness of the program. Data collection should include crash and RLR data; the Department of Transport (2007) recommends that other information, such as public opinion regarding RLCs, also be collected. The FHA (2005) recommends collecting data at both camera locations and control sites without photo enforcement to measure the effects of the RLC program absent other external factors.

### 5.7 Economics

Clearly, the various costs and benefits of operating an RLC program are key considerations for local communities and there are a variety of economic analyses that may be conducted. The most basic is a simple estimate of the costs required to implement and operate a program. Because an RLC program generates revenue in the form of fines (resulting from tickets issued to drivers who run red lights), communities may also be concerned about the program's fiscal viability, which involves comparing implementation and operation costs to the revenue generated by the program. Additionally, analysts may examine the economic impact of changes in the number of traffic crashes.

Many of the studies did not include any economic information; those that did varied widely in the amount of detail provided. Only one report (South et al., 1988) considered monetized cost and crashes, but the brief analysis that was conducted excluded revenue from fines. None of the studies conducted a full cost‐benefit analysis that included both fiscal viability and societal benefits (including crash costs), and there was insufficient comparable information available to permit anything other than a qualitative synthesis of economic information from the included studies.

#### 5.7.1 Implementation and operational costs

Only a small number of studies provided information on implementation costs, which can include factors such as the capital cost of the RLC equipment, the costs involved in setting up an RLC program, and operational or running costs.

According to Fitzsimmons (2007a, 2007b, 2007c) and Hallmark et al., (2010), a typical RLC system can cost US $50,000 or more, depending on both the type of intersection and the number of cameras to be installed. Washington and Shin (2005) also estimated the cost of a 35‐mm wet film camera system to be around US $50,000 to $60,000, including the camera and housing, pole, and loop detectors, as well as installation, and pointed out that digital systems are significantly more expensive, costing up to US $100,000.

Operational costs include the ongoing costs involved in running the RLC program. One of these is the fee charged by the RLC vendor. This can be either a flat monthly fee or a cost per citation issued. Washington and Shin (2005) stated that monthly fees tend to be approximately US $5,000 per camera system and Fitzsimmons (2007a, 2007b, 2007c) and Hallmark et al., (2010) reported similar monthly fees per intersection. Fitzsimmons (2007a, 2007b, 2007c) and Hallmark et al., (2010) also noted that in Iowa, multiple possible payment structures were implemented. While a flat monthly operating fee was one option, cities could elect to pay the vendor a fee per citation. Depending on the vendor, the fee was either fixed or decreased as the number of citations per day increased.

Shin and Washington, (2007; as discussed in Washington & Shin, 2005) suggested that RLC programs could be operated as revenue‐neutral programs, so that the operating costs are equal to the fines generated from the program. However, they also pointed out the difficulty in locating published estimates of installation and operating costs.

#### 5.7.2 Fiscal viability

The studies that included economic information focused either on the program's fiscal viability, which involved comparing implementation and operation costs to revenue generated, or the safety and social benefits of the program, which focused on cost changes resulting from changes in the number of traffic crashes. None of the studies conducted a complete analysis that included both operational costs and societal benefits (including crash costs).

Of the studies that examined fiscal viability, three reported negative economic outcomes (Andreassen, 1995; City of Lubbock, 2009; Sharpnack, 2009). Andreassen (1995) reported capital costs of at least AU $1.8 million and annual running costs of AU $285,000 to operate 15 RLC sites in Sydney with five cameras. Annual revenue in 1989 was AU $272,000, which did not even cover program operating costs. As Andreassen (1995) did not report any significant reduction in crashes from the RLC program, it does not appear that the program was fiscally viable. Sharpnack (2009) found that between 2003 and 2009, the RLC program in Costa Mesa, California raised approximately US $5.7 million. During this period, the city paid approximately US $6 million to the contract vendor, for a net cost to the city of about US $300,000, not counting the cost of civilian police personnel who devoted approximately 30 hr per week to RLC program tasks. Similarly, City of Lubbock (2009) found that between July and December 2007, their program, which included RLCs at 11 intersections, reported a monthly deficit ranging from US $18,858 to US $47,419.94.

Two studies reported both positive and negative economic outcomes when comparing program costs and revenue. California State Auditor (2002) examined six RLC programs in California and reported that three of the programs (Fremont, Oxnard, and San Diego) were generating cumulative net revenues while the other three (Los Angeles, Sacramento, and San Francisco) were operating with a cumulative deficit. The researchers noted that while the vendors provide similar services, they receive different amounts from each local government, which may account for some of the variation in revenue and expenditure. Garber et al. (2005) also found wide variations when they examined the fiscal feasibility of six RLC programs in Virginia. Two programs (Arlington and Fairfax City) reported a net revenue, three (Alexandria, Fairfax County, and Vienna) reported net deficits, and one (Falls Church) was operating at a break‐even point. Fairfax County had the largest annual deficit (an annual net loss of US $97,811) while Arlington had the largest annual gain (an annual net revenue of US $12,499). Overall, the researchers concluded that, in general, the RLC programs were not generating net revenue.

It is interesting to note that RLC programs are often expected to be fiscally viable. This is a unique requirement, as other types of crime prevention programs generally are not held to this standard. Obviously, developing a revenue‐neutral program is desirable given the budgetary constraints faced by police departments today. However, it is not clear why a program needs to break even or actually produce any positive economic outcomes to be considered worthwhile if it produces a significant increase in road safety. The measure of success for a crime prevention program is usually its ability to prevent crime, not to pay for itself.

#### 5.7.3 Crash safety benefits

Five studies (Garber, 2007; Persaud et al. 2005, as discussed in Council et al., 2005; Ross & Sperley, 2011; Shin & Washington, 2007; South et al., 1988) examined the economic effect of changes in the number of traffic crashes that resulted from the use of RLCs, separate from the installation and operational costs of the programs.

Ross and Sperley (2011) examined the overall cost of crashes at one intersection in Salem, Oregon before and after an RLC was installed and found that the average monthly cost of crashes increased by about 70% during the post‐installation period, rising from US $16,296 to US $27,738. They noted that during the period prior to camera installation there was a higher percentage of injury crashes and that most of the crashes occurring after the cameras were installed were rear end crashes, which tend to produce less severe injuries. However, despite this, the overall increase in the average number of crashes per month after the cameras were installed resulted in an overall higher monthly cost.

Garber (2007) focused on the safety impact of cameras in the six programs in Virginia. They reported that injury crashes increased after camera installation but pointed out that not all crash types are equally severe. To measure the effect of RLCs on net injury severity, they examined comprehensive crash costs, monetizing rear end and right angle injury crashes as well as all crashes (injury and non‐injury) combined. They found that in both cases, cameras were associated with increased crash costs in some jurisdictions and decreased costs in others.

Three studies (Persaud et al., 2005, as discussed in Council et al., 2005; Shin & Washington, 2007; South et al., 1988) found positive economic impacts after RLC implementation. South et al. (1988) reported that crash costs at RLC intersections in Victoria, Australia were 13.8% lower than expected when compared to control sites. This was equivalent to an annual net savings of AU $30,253 per site. Shin and Washington (2007) examined crash costs in Phoenix and Scottsdale, Arizona before and after RLC program implementation, using national crash cost estimates for various types of crashes. They estimated a mean annual safety benefit of US $4,504 for the 10 target approaches in Phoenix and US $684,134 for the 14 target approaches in Scottsdale. The difference is reportedly due to the differential impact of the cameras in the two cities; in Phoenix, the cameras primarily contributed to a decrease in PDO crashes while in Scottsdale, they had a greater impact on reducing injury crashes. Council et al. (2005) conducted cost comparisons on crash changes in seven jurisdictions around the United States. When the jurisdictions were combined, they found an overall economic benefit of about US $39,000 per intersection per year when PDO crashes were included and US $50,000 per intersection per year when PDO crashes were excluded from analysis.

#### 5.7.4 Other economic factors

Early cost‐benefit analysis studies have identified significant benefits for RLCs. In the United Kingdom, Hooke et al. (1996) found that the average fixed cost per site of RLCs was just over £9,200 and average annual costs were over £5,600 per site. The study compared the costs of installing and operating RLCS (including capital costs, maintenance, and prosecution costs) to benefits resulting from crash reduction and fixed penalty revenues and found that RLCs generate significant net benefits to the police and the community as a whole. Seven of the 10 areas examined achieved a net positive return within 1 year of camera installation and all but one had done so after 5 years. Overall, the researchers reported that the return on investment was twelve times the initial investment after five years. Additionally, RLCs released traffic police for other duties; the study suggested that a savings of one percent of traffic officer time in England and Wales was equivalent to a national saving of up to £4million. Similarly, in the United States, the Federal Highway Administration (FHA, 2005) stated that RLC systems “provide an economic benefit of $28,000 to $50,000 at a treated site when considering the economic cost of crashes by crash type.”

## 6 DISCUSSION

### 6.1 Summary of main results

This systematic review shows that RLCs can be effective in reducing some types of traffic crashes, particularly right angle crashes, right angle injury crashes, and total injury crashes. They may also reduce red light violations. However, RLCs also appear to be linked to an increase in rear end crashes. There was no evidence to suggest that study heterogeneity was consistently explained by either country or risk of bias. The presence or absence of warning signs did not appear to have an impact on RLC effectiveness. There was some evidence that studies accounting for RTM report more moderate decreases for right angle injury crashes than studies not accounting for RTM. Some evidence suggested when stratified by risk of bias according to control for confounders, studies with a low risk of bias reported only a nonsignificant decrease in right angle crashes.

### 6.2 Deterrence and spillover

As noted in the Results section above, a key mechanism by which RLCs may reduce RLR is deterrence, which focuses on the threat of punishment to discourage offending.

#### 6.2.1 Deterrence theory

According to Gibbs (1975), deterrence is “the omission or curtailment of a crime from the fear of legal punishment” (p. 39). Deterrence theory posits that fear of punishment encourages potential wrongdoers to comply with the law. Empirical research examining the effectiveness of traffic lights and traffic regulations on traffic violations suggests that deterrence may result when violation is associated with penalty and the potential for penalty (Cramton, 1969). This suggests that other mechanisms, such as RLCs, may also affect driver behavior and deter drivers from running red lights.

Porter and Berry (1999) conducted a survey of drivers and found that respondents did not believe that the police would catch most red light runners, so they felt there were few consequences for this behavior. However, surveys of communities in which RLC enforcement has been implemented show different results. For example, a survey conducted by Retting and Williams (2000) found that in cities with RLCs, 61% of respondents considered it likely that someone would get a ticket for running a red light in their city, compared with only 46% of respondents in cities without camera enforcement. Additionally, respondents in cities with cameras were more than twice as likely to know someone who had received a traffic ticket for running a red light as those in cities without cameras. RLCs not only increase the risk of punishment, and thus the negative consequences of RLR, but also increase the perception of risk among the general population. This suggests that the mechanism by which RLCs may reduce RLR is deterrence.

It is important to note that deterrence theory assumes that the target behavior is committed intentionally: drivers may deliberately run red lights or may intentionally try to “beat the light” by speeding up when the light turns yellow. In the latter case, even though drivers may not have actually intended to run the red light, they were aware that their attempt to beat the yellow light created the potential to do so. Deterrence assumes that drivers' behavior can be influenced by RLCs; however, some drivers may be unable to stop or clear the junction safely at the end of the green signal phase (during the yellow signal phase), this is referred to as the dilemma zone. This has implications for safety, as driver behavior may be more unpredictable; one driver may choose to stop and become at risk for a rear end collision, while a driver who continues may be at risk of crossing on the red light and prosecution and at greater risk of a collision at the intersection and potential prosecution (Maxwell & Wood, 2006). Enforcement mechanisms that attempt to deter behaviors such as RLR are unlikely to affect situations in which the behavior is committed inadvertently or unintentionally, such as when a driver was unable to see the signal, was inattentive and did not realize that the traffic light was red, or was unable to stop. Therefore, it is likely that RLCs will be more effective in reducing RLR among drivers who deliberately engage in this behavior.

#### 6.2.2 Types of deterrence

There are two main types of deterrence. Specific deterrence focuses primarily on punishing apprehended offenders, with the assumption that they will be deterred from reoffending out of a desire to avoid future punishment. General deterrence focuses on the population at large and assumes that the threat of punishment will deter people from initial law violations. The greater the perception of risk of punishment, the greater the likelihood that general deterrence will be effective. To be effective, traffic enforcement policies need to do both, so that a sanction not only impacts the individual who is being punished but also others who do not directly experience the sanction (Bates, Soole, & Watson, 2012).

As a specific deterrent, RLCs focus on reducing repeat offending by punishing those individuals who offend. Because RLCs are fully automated and operate remotely, without requiring human intervention, drivers who run red lights are more likely to be detected and punished. One purpose of this punishment is to deter these drivers from running red lights again in the future.

General deterrence is achieved through increasing the risk of apprehension and punishment. This may include both the actual/objective risk of being detected and the perceived/subjective risk, which reflects drivers' belief about the likelihood that they will be detected in a violation. Overall, perceived risk appears to be most likely to influence driving behavior (Zaal, 1994). RLCs are designed to create what Belin, Tillgren, Vedung, Cameron, and Tingvall (2010) calls a “feeling of continued surveillance” that suggests a greater risk of apprehension and punishment for running red lights.

In many cases, the primary aim of enforcement strategies such as RLC programs is general rather than specific deterrence ‐ the focus is on preventing red light violations rather than on offender detection (see, e.g., MacLean, 1985; Zaal, 1994). South et al. (1988) suggests that increased enforcement, obtained through the use of RLCs, may deter potential offenders and Retting and Kyrychenko (2002) also found that the effects of RLCs were not limited to only the specific intersections with cameras, providing further support for a general deterrent effect. Retting et al. (1999a, p.173) suggests that “The presence of cameras may promote a general readiness to stop at red lights.”

#### 6.2.3 Spillover: The halo effect

According to Hu et al. (2011, p.277), “A high likelihood of apprehension helps convince motorists to comply with traffic laws.” RLCs are designed to increase the actual risk of apprehension as well as increasing the perception of increased risk, to create a general deterrent effect. However, their impact may not be limited to only those intersections with cameras. RLCs may also create spillover, so that they have a more general effect on driver behavior at signalized intersections, reducing red light violations not only at those locations where cameras are placed but also at surrounding non‐camera intersections. Retting and Kyrychenko (2002, p. 1825) suggests that the potential for positive spillover is a key element of RLC programs because “the goal of highly conspicuous traffic enforcement is to produce generalized changes in driver behavior with respect to traffic safety laws.”

Attitudinal surveys suggest that RLCs do create an overall perception of increased risk. As discussed above, Retting and Williams (2000) found that in cities with RLC enforcement programs, significantly more respondents believed it was likely that someone who runs a red light would get a ticket. Arup Transportation Planning (1992) surveyed drivers in Brisbane, Australia before and after the implementation of an RLC program. He found that prior to the introduction of RLCs, most drivers did not consider it likely that they would be caught disobeying a red traffic light (only 38% of drivers rated their chances of being caught as “likely” or “very likely”); this increased to 52% after the RLC program was implemented. He also stated that a survey in Adelaide, Australia after the implementation of RLCs found that 85% of people surveyed believed that RLCs “will make it more likely for drivers to get caught running a red light” and 72% believed RLCs “would change their driving habits” (p.27).

South et al. (1988) has pointed out that for RLC enforcement to have an effect, motorists must be aware that cameras are in operation. He discussed a study of RLCs in Victoria, Australia in which RLCs were installed and monitored prior to media publicity of the program. During the period when the public was unaware of the program, approximately 300 violators per week were photographed by the RLCs; this decreased to about 20 violators per week after the media began publicizing the RLC program.

To increase the potential for spillover, and therefore increase the effectiveness of the enforcement program, Ross and Sperley (2011) suggests the use of jurisdictional boundary signs warning drivers about the use of photo enforcement mechanisms, rather than posting signs at the specific traffic intersections where cameras are installed. Shin and Washington (2007) examined the effect of RLCs in two cities in Arizona. In Phoenix, warning signs were placed at all approaches at RLC intersections, while in Scottsdale, signs were only placed on some of the intersections where cameras were deployed. The researchers found significantly greater spillover effects in Scottsdale; they suggested that the drivers in Phoenix knew which intersections and approaches had cameras and modified their behavior accordingly, while drivers in Scottsdale were not as certain of where the cameras were placed and therefore were more likely to be deterred from traffic violations citywide.

In general, empirical tests of the spillover effect of RLCs have yielded inconsistent results. Retting and Kyrychenko (2002) and Retting et al. (1999a) found red light violations in Fairfax and Oxnard decreased at both camera and non‐camera intersections; they argued that in both cities the decline at non‐camera sites was due to spillover (see e.g., Retting, Williams, Farmer, & Feldman, 1999b). Similarly, Kull (2014) found a decline in right angle crash rates in Chicago at intersections with and without RLCs (36% and 27%, respectively), and concluded that the decrease in crashes at the non‐camera sites was due to spillover. AECOM Canada, Ltd. (2014) reported relatively large spillover effects as well, suggesting that the reduction in collisions at non‐RLC intersections was due to changes in driver behavior that may have resulted from the widespread publicity of the RLC program throughout the jurisdiction as well as the public's lack of knowledge as to which specific intersections were equipped with cameras. Chin (1989) found that the installation of RLCs at intersections in Singapore not only reduced RLR on those approaches covered by the cameras but on other approaches as well.

Conversely, while Hobeika and Yaungyai (2006) found a spillover effect of RLCs on PDO crashes in Fairfax, they also concluded that there was no spillover effect for injury crashes. Garber (2007) found that there were no significant changes in various types of traffic crashes at non‐camera sites in Virginia that were used to test for spillover.

### 6.3 Comparisons with the previous Cochrane review

The original systematic review (Aeron‐Thomas & Hess, 2005) included only 10 studies which were all published during 2002 or earlier and which came from only three countries (the United States, Australia, and Singapore). This updated review has expanded the search to a more comprehensive list of databases and websites and has increased the number of included studies to 38. Additionally, the research located by this updated search tends to be of higher quality. The updated review includes four studies that have been classified as high quality, while the original review had none; conversely, 80% of the previously identified studies were of low quality, compared to 64% of the newly identified studies.

The increased number of studies included in the updated review, combined with the addition of a more extensive meta‐analysis, has increased the precision of the effect estimates. The findings of this updated review support those of the original review in some areas, but not in all. As reported in the original review, the updated review found that RLCs were effective in reducing total injury crashes. However, while the original review did not find any significant effect of RLCs on various types of crashes, the updated review did report differential effects of RLCs on specific crash types. RLCs were found to be associated with a reduction in right angle crashes and right angle injury crashes and with an increase in rear end crashes.

Additionally, the updated review has incorporated the EMMIE coding system. Although the original review discussed effect sizes, the other dimensions of the EMMIE scheme were not addressed. In contrast, the updated review includes discussions of the underlying mechanisms, potential moderators, and implementation factors that may influence RLC effectiveness, and economic costs and benefits of RLC programs. EMMIE coding additionally found that while spillover (or diffusion of benefits) is reported in a number of studies, the magnitude of this effect is not established and factors that trigger the underlying mechanism of general deterrence (e.g., warning signs and publicity campaigns) have not been featured widely when devising measures of effectiveness of RLC programs. Practitioners must undertake careful implementation of schemes to ensure legal compliance. Full cost‐benefit analysis including capital, maintenance, operational costs, and revenue alongside societal costs and benefits (including costs or savings associated with increases or decreases in crashes) are lacking in the current literature.

### 6.4 Comparisons with prior meta‐analyses

Two previous meta‐analyses were conducted on the effects of RLCs on traffic crashes (Erke, 2009; Høye, 2013; note that Høye was an update and replication of Erke). A comparison of the results of this study with the prior meta‐analyses was inconsistent.

When including all studies, regardless of quality level, both this study and Erke (2009) reported a nonsignficant decrease in total crashes associated with RLCs while Høye (2013) found a nonsignficant increase. Total injury crashes cannot be compared because the earlier meta‐analyses analyzed injury and fatal crashes separately while this study merged the two. This study and Høye (2013) both observed a small nonsignificant increase in PDO crashes while Erke (2009) reported no effect of RLCs on this type of crash.

All three studies found that RLCs were associated with a decrease in right angle crashes (nonsignificant in Høye, 2013); similarly, right angle injury crashes were found to decrease in all three studies, although this result was nonsignificant in Erke (2009). In all three meta‐analyses, rear end crashes increased with the use of RLCs (nonsignificant in Erke, 2009), as did rear end injury crashes (significant in Erke, 2009 only).

Høye (2013) suggested some evidence that warning signs improve total crash outcomes when used with RLCs. However, this study did not find any consistent pattern or significant differences between those studies that reported the use of warning signs and those that did not.

Both Erke (2009) and Høye (2013) found that those studies that controlled for RTM reported less favorable effects of RLCs; this study found some evidence of this when examining right angle and right angle injury crashes. Erke (2009, p.903) suggests that failing to control for RTM “can lead to an overestimation of the effects of RLCs.” Erke (2009) also reported the likely presence of other moderators of effects of RLC that could not be accounted for in a meta‐analysis, similar to the findings in this research.

Overall, the comparison of this research with the previous meta‐analyses suggests that future research needs to look specifically for other possible factors that may cause RLCs to increase or decrease crashes at particular intersections.

### 6.5 Limitations of the review process

One limitation of this review is the lack of high‐quality studies assessing the effectiveness of RLCs. There were no RCTs. While the number of studies in the updated review is almost four times that of the original review, only five of these studies were assessed as having a low risk of bias across all six domains. Two‐thirds of the studies examined in the updated review were assessed as having a high risk of bias across more than one domain. There is limited geographic coverage, particularly among the studies with lowest risk of bias. All five of these studies were conducted in the south‐eastern portion of the United States (two in Florida, two in North Carolina, and one in Virginia). The original review included research from only three countries (the United States, Australia, and Singapore). Aside from the addition of two studies conducted in Canada (AECOM Canada, Ltd., 2014; Sayed & de Leur, 2007), the updated review was still limited to research conducted in these countries. Additionally, the lack of methodological rigor, particularly the failure of many studies to account for both spillover and regression to the mean, and of some to control for additional confounders affects the quality of the evidence in those studies.

The limited geographical coverage is particularly limiting for practitioners looking for insight into the use of RLCs in other countries. Roadways in the United States or Australia differ in various ways from those in the United Kingdom or in Europe. This means that practitioners should be careful when generalizing the results to other countries.

Another limitation was that not all of the included studies examined all of the outcome measures of interest. Most noticeably, only a small number of studies examined the impact of RLCs on RLR or on PDO crashes. The operationalization of the outcome measures also varied; for example, it was not always clear whether or not “possible injury” crashes were included in injury crash counts. Not all studies clearly defined the various crash types, making comparisons more difficult. Importantly, due to the limited occurrences of fatal crashes and more severe injury crashes, few studies reported these as separate outcomes, although they are likely to be of increased interest in terms of crash and injury reduction.

Additionally, the results of the meta‐analysis may be affected by the high levels of heterogeneity found among the results of the included studies. The measure of heterogeneity was significant for all main outcome measures except red light violations. This means that the effect sizes within a given outcome lacked consistency. While subgroup analyses were carried out, in most cases heterogeneity remained high; the only exception was that for some outcome measures, studies conducted in Australia showed little heterogeneity and strong agreement regarding effect sizes.

## 7 AUTHORS' CONCLUSIONS

### 7.1 Implications for practice and policy

Several implications for criminal justice professionals, policymakers, and others involved in traffic engineering and enforcement have emerged from this research.

Consideration needs to be given regarding whether the benefits of RLCs outweigh the costs. This review suggests that RLCs are an effective method for reducing some types of traffic crashes, including total crashes involving injury, and that they may also reduce red light violations. Given continued increases in traffic volume and increasingly‐limited police budgets, which restrict the amount of time police are able to devote to traffic enforcement, RLCs appear to be a viable way of protecting public health and safety. However, RLCs also are associated with an increase in rear end crashes. The potential benefits of a reduction in traffic violations and in some types of injury crashes should be weighed against the increased risk of other crash types.

In addition, policymakers must consider the economic implications of operating an RLC program, including not only the basic costs of installation and operation but also the economic impact of the effects that RLCs have on traffic violations and traffic crashes.

Policymakers must also consider public concerns about the possibility that RLCs may invade motorists' personal privacy, both because RLC data may be accessible to third‐party vendors and because of possible misuse of RLC data by the criminal justice system. Legal challenges to RLC enforcement over these issues as well as possible due process violations may create significant obstacles to the implementation of RLC programs.

### 7.2 Suggestions for future research

Researchers attempting to design high‐quality evaluations of RLC programs should consider the results of this review. A number of the studies examined in this review were limited by methodological weaknesses, including a failure to adjust for the effect of spillover or regression to the mean and a lack of adequate control for confounders. Future research clearly should address these factors. The use of RCTs, something that has not been conducted as yet, also would be an advantage for future studies. Additionally, when possible, researchers should control for the possible moderators identified in this review. The effects of variations in the implementation of RLC programs also should be considered when evaluating such programs.

Researchers examining traffic crash reduction programs generally focus primarily on the most serious types of crashes, particularly those involving fatalities and/or severe injuries. Research on PDO crashes and crashes resulting in less serious injuries is limited. Additionally, because the most serious types of crashes are fairly rare, the current research often merges together crashes of varying levels of severity. Future research should focus on distinguishing between fatal/severe injury crashes and minor injury/PDO crashes.

In some countries, particularly the United Kingdom, safety cameras, which enforce both red light and speed limit violations, are used, increasing the possible safety benefits at high‐risk junctions. This review excluded research into the effects of safety cameras, because it is impossible to separate out the effects of speed versus red light enforcement. Future research should examine the effects of these combined safety cameras to determine if they provide additional road safety benefits over and above those resulting from RLCs.

Both the US and UK guidelines for the implementation of RLC programs emphasized the use of appropriate warning signs when deploying RLCs. However, very few of the studies examined the impact of such signage on the effectiveness of RLCs. Future research should explore the effect of various types and locations of warning signs on RLR and on different types of crashes.

Similarly, both the US and UK guidelines stress the need to publicize RLC programs and to develop educational programs to educate the public about the dangers of red light violations and the ways in which RLCs may enhance traffic safety. None of the included studies specifically examined the impact of publicity or educational programs on RLC effectiveness, although many emphasized the importance of such programs. Future research should evaluate various types of publicity programs and educational strategies on the effectiveness of RLC programs.

## ROLES AND RESPONSIBILITIES

- Content: Ellen G. Cohn, Suman Kakar, Chloe Perkins.
- Systematic review methods: Ellen G. Cohn, Suman Kakar, Phil Edwards.
- Statistical analysis: Ellen G. Cohn, Suman Kakar, Chloe Perkins, Rebecca Steinbach, Phil Edwards.
- Information retrieval: Ellen G. Cohn, Suman Kakar, Chloe Perkins, Phil Edwards.

## SOURCES OF SUPPORT

External: The UK team received funding from the College of Policing and the Economic and Social Research Council (ESRC); Grant title: 'University Consortium for Evidence‐Based Crime Reduction'. Grant Ref: ES/L007223/1.

Internal: None.

## CONFLICT OF INTERESTS

The authors declare that there are no conflict of interests.

## PRELIMINARY TIMEFRAME

Approximate date for submission of the systematic review: December 31, 2018.

## PLANS FOR UPDATING THE REVIEW

This review will be updated on a 5‐year basis. This will require identifying and coding any new studies and rerunning the analyses.

## Figures and Tables

**Figure H1:**
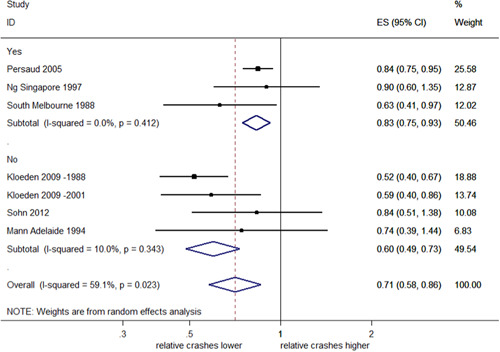
Effects of red light cameras on right angle crashes resulting in injury—stratified by whether or not studies account for regression to the mean (*Q *= 7.78, *p *= .01). CI, confidence interval; ES, effect size

**Figure H2:**
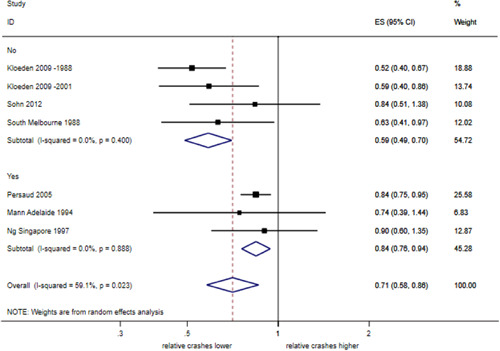
Effects of red light cameras on right angle crashes resulting in injury—stratified by whether or not studies were peer reviewed prior to publication (*Q *= 11.50, *p *= .001). CI, confidence interval; ES, effect size

**Figure H3:**
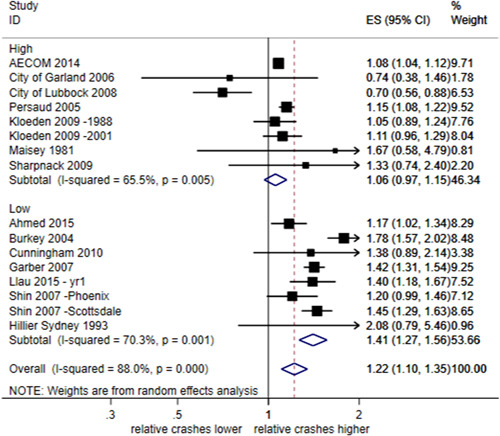
Effects of red light cameras on rear end crashes—stratified by risk of bias according to control for confounders (*Q *= 17.11, *p *< .001)

**Figure H4:**
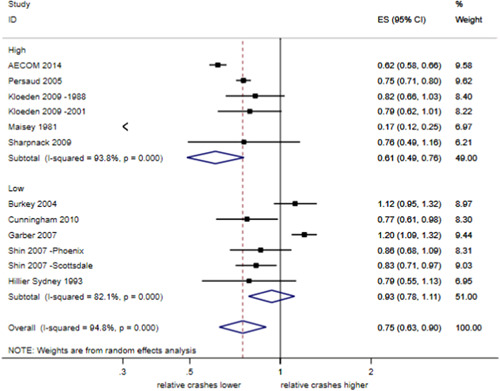
Effects of red light cameras on right angle crashes—stratified by risk of bias according to control for confounders (*Q *= 9.00, *p *= .003). CI, confidence interval; ES, effect size

**Figure H5:**
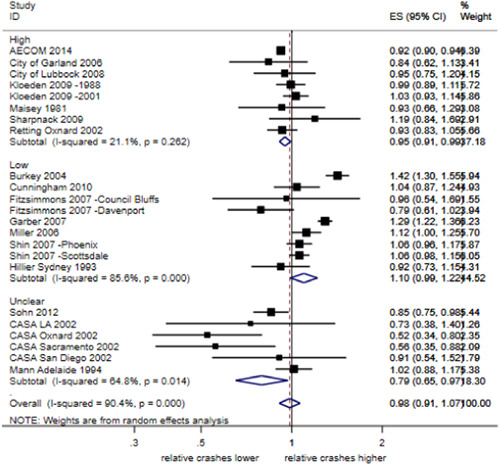
Effects of red light cameras on total crashes—stratified by risk of bias according to control for confounders (*Q *= 0.98, *p *= .01). CI, confidence interval; ES, effect size

**Figure H6:**
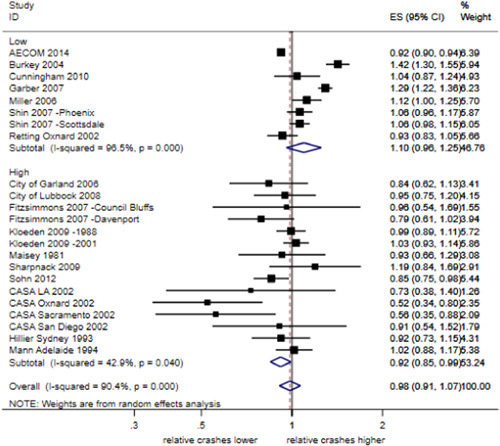
Effects of red light cameras on total crashes—stratified by risk of bias according to control for any other potential sources of bias (including RTM and spillover; *Q* = 5.03, *p *= .02). CI, confidence interval; ES, effect size; RTM, regression to the mean

## APPENDIX A ELECTRONIC DATABASES SEARCHED IN THIS STUDY

The Cochrane Injuries Group Trials Search Co‐ordinator (DB) searched the following:

- Cochrane Injuries Group Specialised Register (to 16 March 2016)
- Cochrane Library CENTRAL database (to 16 March 2016)
- Ovid MEDLINE(R), Ovid MEDLINE(R) In‐Process and Other Non‐Indexed Citations, Ovid MEDLINE(R) Daily and Ovid OLDMEDLINE(R) 1946 to 16 March 2016
- Embase Classic+Embase (OvidSP) 1947 to 16 March 2016
- ISI WOS: SCI‐EXPANDED (1970) and CPCI‐S (1990) to 16 March 2016
- OVID Transport database (1988 to June 2015)—updated March 2016

The librarian at the National Police Library searched the following:

- PROQUEST (to 12 June 2015)
- EBSCO (to 12 June 2015)
- Web of Knowledge (to 12 June 2015)
- Heritage (to 12 June 2015)
- National Police Library (to June 2015)

The Florida International University research group (E. G. C. and S. K.) searched the following (all to 12 June 2015):

- Criminal Justice Abstracts
- Dissertation Abstracts
- EconLit
- EThOS (UK E‐Theses Online Service)
- Health and Safety Science Abstracts
- Hein Online
- Interuniversity Consortium for Political and Social Research (ICPSR)
- JSTOR
- LexisNexis: Academic
- National Criminal Justice Reference Service (NCJRS)
- PsycINFO
- ProQuest CJ
- Sociological Abstracts
- Transport Research and Innovation Portal (TRIP)
- Web of STRID—an integrated database combining records from the Transportation Research Board's Transportation Research Information Services (TRIS) Database and the Organization for Economic Cooperation and Development's Joint Transport Research Centre's International Transport Research Documentation SITRD database
- Web of Science

## APPENDIX B SPECIALIZED WEBSITES SEARCHED IN THIS STUDY

The Florida International University research group searched the following websites (all during February 2016 unless otherwise noted)

- AA Foundation for Traffic Safety, USA—www.aaafoundation.org
- American Transportation Research Institute—http://atri‐online.org/
- Australasian College of Road Safety—http://acrs.org.au/ (searched June 2017)
- Australia and New Zealand Society of Evidence Based Policing—http://www.anzsebp.com/
- Australian Transport Safety Bureau (ATSB)—https://www.atsb.gov.au
- Belgian Road Safety Institute—http://www.ibsr.be/en
- Center for Evidence‐Based Crime Policy, USA—http://cebcp.org/evidence‐based‐policing/
- Center for Problem‐Oriented Policing, USA—http://www.popcenter.org/
- Centers for Disease Control and Prevention (CDC), USA—http://www.cdc.gov/
- Centre for Accident Research and Road Safety (CARRS‐Q), Queensland, Australia—https://research.qut.edu.au/carrsq/ (searched June 2017)
- Chalmers University of Technology, Area of Advance Transport, Sweden—http://www.chalmers.se/en/areas‐of‐advance/Transport/Pages/default.aspx
- CROW, Netherlands—http://www.crow.nl/english‐summary
- Department of Transport Planning and Engineering, National Technical University of Athens, Greece—http://www.civil.ntua.gr/departments/transport/
- European Road Safety Observatory (ERSO)—http://ec.europa.eu/transport/wcm/road_safety/erso/index-2.html
- European Transport Safety Council—http://etsc.eu
- Federal Highway Administration (FHWA), USA—http://www.fhwa.dot.gov/
- Federal Highway Research Institute (BASt), Germany—www.bast.de/EN/Home/
- Finnish Transport Agency — http://portal.liikennevirasto.fi/sivu/www/e
- French Institute of Science and Technology for Transport, Development, and Networks (IFSTTAR)—http://www.ifsttar.fr/en/welcome/
- Institute for Road Safety Research (SWOV), Netherlands—http://www.swov.nl/index_uk.htm
- Institute for Transport Sciences (KTI), Hungary—http://www.kti.hu/index.php/home
- Institute of Transport Economics (TOI), Norway—https://www.toi.no/?lang=en_GB
- Institute of Transportation Engineers (ITE), USA—http://www.ite.org/
- Institute of Transport Studies (Monash), Australia—http://www.eng.monash.edu.au/civil/research/centres/its/
- International Transport Forum—http://www.internationaltransportforum.org/
- Justice Research and Statistics Association, USA—http://www.jrsa.org
- Laboratoire d'Economie des Transports (LET), France—http://www.let.fr/?lang=en
- National Highway Traffic Safety Administration (NHTSA), USA—http://www.nhtsa.gov/
- Police Executive Research Forum, USA—http://www.policeforum.org/
- Police Foundation, USA—http://www.policefoundation.org/
- Rand Corporation, USA—http://www.rand.org/
- Scottish Institute for Policing Research—http://www.sipr.ac.uk/
- Society of Evidence Based Policing, UK—http://www.sebp.police.uk/
- Swedish National Road and Transport Research Institute (VTI)—http://www.vti.se/en/
- Swiss Council for Injury Prevention (BFU)—http://www.bfu.ch/en
- Technion – Israel Institute of Technology—http://www.technion.ac.il/en/
- Trafikverket/The Swedish Transport Administration—http://www.trafikverket.se/en/startpage/
- Transport Canada (TC)—www.tc.gc.ca/eng/
- Transport Research Board (TRB), USA—http://www.trb.org
- Transport Research Center (CDV), Czech Republic—http://www.cdv.cz/en/
- Transport Research Laboratory (TRL), UK—http://trl.co.uk/
- Transport Safety Research Centre (TSRC), UK—http://www.lboro.ac.uk/departments/lds/research/groups/tsrc/
- United Nations Official Documents System—http://documents.un.org/
- Vera Institute of Justice, USA—http://www.vera.org/
- VTT Technical Research Centre of Finland, LTD (VTT)—http://www.vttresearch.com/
- World Health Organization (WHO)—http://www.who.int/en/

## APPENDIX C FULL SEARCH STRATEGY (AS USED FOR MEDLINE)

1. Photography/
2. (enforc* adj3 program*).ab,ti.
3. Law Enforcement/
4. Social Control Policies/
5. ("road safety" adj3 legislation).ab,ti.
6. ("red light" adj5 (running or camera*)).ab,ti.
7. camera*.ti,ab.
8. (camera* adj5 (traffic or intersection* or junction* or automat*)).ab,ti.
9. (speed adj5 (camera* or control or enforc* or limit* or measurement*)).ab,ti.
10. ((photo* or automat*) adj3 enforc*).ab,ti.
11. (radar adj3 gun).ab,ti.
12. (radar adj3 (gun* or laser*)).ab,ti.
13. 1 or 2 or 3 or 4 or 5 or 6 or 7 or 8 or 9 or 10 or 11 or 12
14. "Wounds and Injuries"/
15. Accidents, Traffic/
16. ((accident* or colli* or fatal* or injur* or crash* or speed*) adj3 (traffic or road or vehicle or automobile)).ab,ti.
17. 14 or 15 or 16
18. 13 and 17

## APPENDIX D EPPI REVIEWER STANDARDIZED DATA CODING SET FOR EMMIE FRAMEWORK

**Table:**

Mechanism	Were any of these mechanisms mentioned? Were any mentioned mechanisms tested? Were any intermediate outcome measures taken to assess the activation of the causal mechanism(s) judged to underpin the intervention?
Moderators	What was the country of implementation? What was the setting of the intervention? What were the road and traffic characteristics? Was the intervention implemented in isolation? Was it stand‐alone, as part of a package or unclear? Was monitoring/intervention undertaken at specific times? ‐times of day, season or constant What type of technology was used?
Implementation	What (if any) implementation factors were mentioned? Which (if any) implementation factors were stated as necessary for success? Who was responsible for deciding the eligibility and location of intervention sites? Who was responsible for setting up operating/maintain/ticketing intervention? What (if any) obstacles to implementation existed? What resources were required? What personnel requirements (training, etc.) existed?
Economics	What was the cost of the program set‐up? What were the annual running costs of the program? What is the cost of a red light camera? What is the cost of a crash? What are the personnel/hours costs? What costs are related to direct and indirect benefits? What is the cost‐benefit ratio? (Cost‐benefit analysis)

## APPENDIX E STANDARDIZED DATA EXTRACTION FORM

**Table:**

Queries	Responses
Study	
Short name of study (first author/date)	
Linked studies (if any)	
Location (city/country)	
Methodology	
Participant junctions	
Interventions (RLCs)	
Outcome measures	
Signage/publicity (if any)	
Distance used for identifying RLC crashes	
Method of identifying RLR	
Notes	
Final decision (include/exclude)	

## APPENDIX F CHARACTERISTICS OF STUDIES

### Included studies

Description of 28 newly identified studies (with additional subsumed studies where appropriate).

AECOM Canada, Ltd. (2014; Alberta, Canada)

**Table:**

Methods	CBA with Empirical Bayes analysis. Study period 1995–2008, RLC implementation dates varied between municipalities
Participants	217 signalized and 47 unsignalized intersections across 5–7 municipalities in Alberta
Interventions	76 signalized intersections with RLCs, 141 non‐camera signalized intersections, 47 non‐camera unsignalized intersections
Outcomes	Total crashes, property damage‐only crashes, right angle crashes, rear end crashes
Notes	

Ahmed and Abdel‐Aty (2015; Florida, USA)

**Table:**

Methods	CBA with Empirical Bayes analysis, minimum of 3 years before and 3 years after period
Participants	75 signalised intersections in Florida, USA
Interventions	25 RLC equipped intersections, 50 adjacent non‐camera spillover sites
Outcomes	Total right angle and left turn crashes, total rear end crashes, injury right angle and left turn crashes, injury rear end crashes
Notes	Safety performance function was calculated using 950 signalized intersections across Florida. Data were extracted only for intersection‐related crashes, and crashes associated with drugs and alcohol were excluded. Data presented “target approaches” at intersections and “all approaches”

Andreassen (1995; Melbourne, Australia)

**Table:**

Methods	CBA with 5 years before data (1979‐1983) and 5 years after data (1985–1989)
Participants	All signalized intersections in the Melbourne metropolitan area.
Interventions	41 RLC sites (installed in 1984), crashes at all signalized Melbourne Metropolitan intersections used as comparison
Outcomes	Pedestrians hit crossing roads, right angle crashes, rear end
Notes	Selection of sites was from a list of signals ranked by right angle casualty crashes. 75% of sites had initial average frequencies of 2 or less crashes/year Total crashes used in comparison includes the crashes at RLC sites

Arup Transportation Planning (1992; Brisbane, Australia)

**Table:**

Methods	CBA. Before data collected between November 19 and 30, 1990, after data collected between November 27 and December 10, 1991. Data collected during peak (7.30–9.30 am and 4–6 pm weekdays) and off peak (all other times) hours
Participants	12 signalized intersections around Brisbane
Interventions	6 RLC sites, 6 non‐camera control sites
Outcomes	Red light violations
Notes	Before/after data periods very short

Burkey and Obeng (2004; North Carolina, USA)

**Table:**

Methods	Controlled interrupted time series over 57 months, including at least 26 months of before data and 22–31 months of after data
Participants	303 signalized intersections in Greensboro, North Carolina
Interventions	18 RLC equipped intersections, 285 non‐camera signalized control intersections
Outcomes	Total crashes, severe injury crashes, possible injury crashes, property damage‐only crashes, right angle crashes, left turning different roadway crashes and rear end crashes
Notes	Crashes only included if they result in either an injury or USD $1,000 worth of property damage

Chin (1989; Singapore)

**Table:**

Methods	CBA. Before data collected between January 19 and February 17, 1987, after data collected between April 14 and May 26, 1987 for 1 day at each intersection.
Participants	16 intersections in Singapore, with appropriate vantage point (e.g., tall building) from which to operate video camera of intersection
Interventions	11 RLC intersections and 5 non‐camera intersections
Outcomes	Red light violations
Notes	Before/after data periods very short. One day survey at each intersection only, data only collected on dry weekdays

City of Garland (2006; Texas, USA)

**Table:**

Methods	CBA with 31 months before data and 31 months after data, RLCs implemented on September 16, 2003
Participants	4 high volume intersections in Garland, Texas
Interventions	4 RLC intersections with wet film cameras on one approach road, 6 similar control intersections in Garland
Outcomes	Total crashes, red light runner total crashes, rear end crashes
Notes	

City of Lubbock (2008; Texas, USA)

**Table:**

Methods	CBA with data collected Jul–Dec for 2005, 2006, and 2007. Two periods of 6 months before data (Jul–Dec 2005 and 2006), and one 6‐month period of after data (Jul–Dec 2007)
Participants	17 intersections in Lubbock City Council, Texas.
Interventions	11 RLC intersections, 6 non‐camera comparison intersections
Outcomes	Total crashes, RLR crashes (resulting from red light violations), rear end crashes, total injury crashes.
Notes	Based on a City Council Work session presentation with few study details.

Persuad (2005; also Council et al., 2005; CA, MD, NC, USA)

**Table:**

Methods	CBA with Empirical Bayes analysis. Average before period of 6 years and average after period of 2.76 years
Participants	836 intersections across 7 jurisdictions around the USA (El Cahon, San Diego, San Francisco, CA; Howard County, Montgomery County and Baltimore, MD; and Charlotte, NC) with identified RLC problems in mid‐late 1990s
Interventions	132 RLC treatment sites (between 6 and 31 in each jurisdiction), 296 unsignalized non‐camera comparison sites (around 40–50 in each jurisdiction) similar to RLC sites and 408 signalized non‐camera reference sites (to calibrate safety performance functions and investigate spillover, 34–86 in each jurisdiction)
Outcomes	Total right angle crashes (right angle, broadside or turning crashes from perpendicular approaches), right angle definite injury crashes (exclude “possible” injury), rear end crashes, rear end definite injury crashes
Notes	Injury crashes include fatal and definite injury, exclude possible injury crashes

Cunningham and Hummer (2004; North Carolina, USA)

**Table:**

Methods	CBA with 5 years of before data and less than one year of after data
Participants	24 signalized intersections in Raleigh, North Carolina
Interventions	12 intersections equipped with RLCs
Outcomes	Total crashes, angle crashes, rear end crashes
Notes	Results may be affected by seasonality as there is not a full year of data in the after period
	Comparison group with halo effect methodology also included to control for spillover (using subset of comparison sites on same roadway as treated sites)

Cunningham and Hummer (2010; North Carolina, USA)

**Table:**

Methods	CBA with 5 years of before data and 46 months of after data
Participants	14 intersections in Raleigh, North Carolina
Interventions	6 intersections equipped with RLCs
Outcomes	Total crashes, angle crashes, rear end crashes, red light running related crashes (sum of right angle, rear end and left turn crashes), injury crashes
Notes	Comparison group with halo effect methodology also included to control for spillover (using subset of comparison sites on same roadway as treated sites)

Fitzsimmons (2007a, 2007b; Council Bluffs and Davenport, Iowa, USA)

Council Bluffs

**Table:**

Methods	CBA with variable amounts of before and after data
Participants	9 signalized intersections in Council Bluffs, Iowa
Interventions	5 RLC intersections (7 intersection approach roads)
Outcomes	Total crashes, rear end crashes, RLR crashes (identified from crash report as ran a red signal)
Notes	Further cross sectional analysis in Clive, Iowa not extracted

Davenport

**Table:**

Methods	CBA with Empirical Bayes analysis, variable amounts of before and after data
Participants	9 signalized intersections in Davenport, Iowa
Interventions	4 RLC intersections (6 intersection approach roads)
Outcomes	Total crashes, rear end crashes, RLR crashes (identified from crash report as ran a red signal)
Notes	Further cross sectional analysis in Clive, Iowa not extracted

Hallmark, et al. (2010; Davenport, Iowa, USA)

**Table:**

Methods	CBA with Empirical Bayes analysis, variable amounts of before and after data
Participants	9 signalized intersections in Davenport, Iowa
Interventions	4 RLC intersections (6 intersection approach roads), cameras installed in 2004, 5 non‐camera control intersections in Davenport with similar traffic volumes, crash frequencies and roadway types
Outcomes	Total crashes, RLR crashes, RLR rear end crashes (identified from crash reports as ran a red signal)
Notes	

Garber (2007; also Garber et al., 2005; North Virginia, USA)

**Table:**

Methods	CBA with Empirical Bayes analysis of 3500 crashes over 7 years
Participants	72 signalized intersections over 6 jurisdictions in Northern Virginia
Interventions	28 RLC intersections and 44 control intersections, with an additional 15 spillover sites (within 0.6 miles of RLC intersection)
Outcomes	Total crashes, injury crashes, angle crashes, rear end crashes, RLR crashes (driver charged with RL violation), injury RLR crashes
Notes	Employed 4 difference analyses with only EB extracted (paired *t* test, analysis of variance, generalized linear modelling and Empirical Bayes)

Hobeika and Yaungyai (2006; also Yaungyai, 2004; Fairfax County, Virginia, USA)

**Table:**

Methods	CBA with 1–2 years of before data and 1–2 years of after data
Participants	14 intersections in Fairfax County, Virginia
Interventions	13 RLC intersections, 4 non‐camera control intersections (away from RLC zone with high numbers of crashes)
Outcomes	Total crashes, fatal crashes, injury crashes, property damage‐only crashes
Notes	(Yaungyai, 2004 is MSc Thesis by 2nd author). No appropriate data for extraction, with some unexpected % changes, e.g., Table VI PDOS at site 9, would require more data to include

Hu et al. (2011; USA)

**Table:**

Methods	CBA citywide analysis, before data period between 1992 and 1996 and after data period between 2004 and 2008.
Participants	62 large cities (>200,000 residents in 2008) with and without RLC programs in USA
Interventions	14 cities identified with RLC enforcement for all of after period and none of before, 48 cities identified with no RLC enforcement in before or after periods
Outcomes	Citywide per capita rate of fatal red light running crashes (one driver assigned “failure to obey traffic control device” in crash report), citywide per capita rate of all fatal crashes at signalized intersections
Notes	

Kloeden et al. (2009; 1988 and 2001; Adelaide, Australia)

**Table:**

Methods	CBA with 4 years before and 4 years after data for RLCs installed in 1988 and 1 year before and 1 year after data for RLCs installed in 2001
Participants	All signalized intersections in Adelaide metropolitan area from mid‐1988
Interventions	6 RLCs rotated among 8 signalized intersections from 1988; 24 signalized intersections with RLCs added in 2001. Comparison with all other signalized intersections in Adelaide (1983–2002)
Outcomes	Total crashes, total injury crashes, right turn crashes, right turn injury crashes, right angle crashes, right angle injury crashes, rear end crashes, rear end injury crashes, other injury crashes
Notes	

Ko et al. (2013; Texas, USA)

**Table:**

Methods	CBA with Empirical Bayes analysis. Before data collected between 1 and 4 years before and after RLC implementation. Total of 516 and 663 intersection years before and after data, respectively
Participants	311 signalized intersections across 32 jurisdictions in Texas
Interventions	RLCs at 245 signalized intersections, 66 reference non‐camera intersections (at least 2 miles from treated site to minimize spillover)
Outcomes	Total RLR crashes, rear end RLR crashes, right angle RLR crashes (RLR crashes identified from crash reports if it included one of following factors: disregard of stop‐go signal, failure to yield the right‐of‐way during a turn on red)
Notes	Outcomes are all specific to RLR crashes identified from crash reports (not crash type)

Kull (2014; Chicago, Illinois, USA)

**Table:**

Methods	CBA conducted between 2005 and 2012 with minimum of 1 years before and 1 year after data. Median RLC “go live” data of July 31, 2007, use for before/after distinction for control group
Participants	All signalized intersections in Chicago
Interventions	170 RLC signalized intersections, all other signalized non‐camera intersections in Chicago used as control
Outcomes	Angle injury crashes, rear end injury crashes, turning injury crashes, total injury crashes
Notes	Changes in reporting in 2009 raised the minimum cost for PDO reporting from $500 to $1,500; therefore, only crashes featuring at least one injury were used

Llau et al. (2015; Florida, USA)

**Table:**

Methods	CBA with Empirical Bayes analysis using 3 years before data (2008–2010) and 2 years after data (2011–2012).
Participants	60 signalized intersections in Miami‐Dade County, Florida
Interventions	20 intersections with RLCs and 40 non‐camera comparison sites. 2 comparison sites matched for each treated site, all at least 2 miles away
Outcomes	Right angle and turning crashes, rear end crashes, all injury crashes (includes possible injuries), injury right angle and turning crashes. (RLR related crashes defined as the sum of right angle and turning crashes)
Notes	

Maisey (1981; Perth, Australia)

**Table:**

Methods	CBA, with two 1 year periods of before data (Jul 1977–Jul 1979) and 1 year of after data (Jul 1979–Jul 1980).
Participants	10 signalized intersections in Perth
Interventions	1 RLC signalized intersection (4 way approach) and 9 non‐camera, signalized, 4 way control intersections
Outcomes	Total crashes, rear end crashes, right angle and indirect (turning) right angle crashes, injury crashes
Notes	Study also looked separately at total crashes during daylight versus darkness

Miller et al. (2006; also Khandelwal and Garber, 2005; Fairfax County, Virginia, USA)

**Table:**

Methods	CBA with Empirical Bayes analysis. Data collected between 1998 and 2003. RLC installation began in 2000, varied before and after periods with a minimum of 2.8 years before and 0.8 years after
Participants	36 sites in Fairfax County, Virginia
Interventions	13 RLC sites and 33 non‐camera sites selected based on recommendations from Virginia Department of Transportation
Outcomes	Total crashes, injury crashes, injury crashes attributable to RLR, rear end crashes attributable to RLR, total crashes attributable to RLR. (A crash was categorized as RLR if collision type was coded as “disregarded stop‐go light” or if the narrative in crash report clearly states a driver ran red light)
Notes	Crash data for RLC sites obtained from routine databases, crash data for comparison sites from manual examination of crash reports forms.

Porter et al. (2013; Virginia, USA)

**Table:**

Methods	CBA with two time points before RLCs switched off and 4 points after switch off
Participants	8 signalized intersections in Virginia
Interventions	4 RLC equipped intersections, 2 non‐camera spillover controls in Virginia Beach, and 2 non‐camera controls in Newport News, Virginia
Outcomes	Proportion of last drivers (those entering the intersection before oncoming traffic) who ran red lights
Notes	This study estimated the effect of turning RLCs off, rather than turning them on, and is therefore not included in the data extraction

Pulugurtha and Otturu (2014; Charlotte, North Carolina, USA)

**Table:**

Methods	CBA with Empirical Bayes analysis. Before data varied from 1.5 to 3.5years, after data varied between
Participants	80 signalized intersections in Charlotte, North Carolina
Interventions	32 RLC intersections and 48 signalized non‐camera intersections (similar to treated intersections)
Outcomes	Total crashes
Notes	Includes some after data for different crash types, only total crashes included in EB analysis and extraction

Retting et al. (1999b; Oxnard, CA, USA)—subsumed in Retting and Kyrychenko (2002) included in previous review

**Table:**

Methods	CBA. Around 24 hr before and 24 hr after data collected for non‐camera sites and between 120 and 241 hr of both before and after data collected at RLC sites
Participants	12 intersections in Oxnard and 2 control intersections in Santa Barbara
Interventions	9 RLCs and 3 non‐camera spillover sites in Oxnard, with 2 non‐camera controls in Santa Barbara
Outcomes	Red light running violations
Notes	Very short before and after periods of data collection. Data collected using video cameras deployed by researchers at sites. Data extracted for control sites

Richardson (2003; Sydney, Australia)

**Table:**

Methods	CBA with Empirical Bayes analysis. RLCs installed between Jan 1988 and Oct 1995, before data collected to 5 years prior to RLC installation through to 2001
Participants	16 intersections in Sydney
Interventions	8 RLC and 8 control intersections. Control sites located close to RLC locations
Outcomes	Annual total crashes, right angle crashes (perpendicular approaches), turning crashes (turning, same roadway), rear end crashes, other crashes
Notes	

Ross and Sperley (2011; Salem, Oregon, USA)

**Table:**

Methods	CBA with 50 months of before data (Jan 2004–Feb 2008) and 21 months after data (Apr 2008–Dec 2009) with RLCs installed March 2008 (excluded from analysis)
Participants	3 intersections in Salem, OR
Interventions	1 RLC intersection (with cameras at 2 approaches), 2 non‐camera intersections nearby
Outcomes	Average monthly total crashes (all within 300 ft of intersection analyzed)
Notes	Other analyses conducted but before and after data only available for average monthly crashes

Sayed and de Leur (2007; also Sayed and de Leur, 2006; Edmonton, Canada)

**Table:**

Methods	Collision prediction models and Empirical Bayes analysis. 3 years of before data and 2–3 years of after data, RLCs implemented between 1999 and 2002
Participants	25 RLC sites, 26 comparison group locations, 100 reference group sites
Interventions	25 RLCs at typical 4 way intersections (3–4 lanes on each approach and 50 km/hr speed limit) 46 non‐camera control sites (with same traffic and environmental conditions as treated sites), some located close to treated sites so could not account for spillover. 100 reference group sites (similar characteristics to treated sites) used to develop collision prediction model
Outcomes	Total crashes, severe crashes (fatal and injury crashes), property damage‐only crashes, right angle head on crashes, rear end crashes.
Notes	Analysis carried out on 25 sites, one site appeared to be an outlier and analysis was also conducted on 24 sites, extracted only data for the evaluation of all 25 sites.

Sharpnack (2009; Costa Mesa, California, USA)

**Table:**

Methods	CBA with before data collected from 2001 to 2003 and after period from 2006 to 2008
Participants	6 intersections in Costa Mesa
Interventions	2 RLCs and 4 non‐camera control intersections
Outcomes	Total crashes, injury crashes, broadside crashes (right angle), rear end crashes
Notes	

Shin and Washington (2007; also Washington, 2005; Arizona, USA)

Four different methodologies used in two cities in AZ; simple before/after study, before/after study with traffic flow correction, before/after study with comparison group and Empirical Bayesian before/after study. CBA method used in Phoenix and CBA with EB in Scottsdale:

Phoenix, AZ—CBA

**Table:**

Methods	CBA, data collected between Oct 1998 and Sept 2003. RLC installation dates and before/after periods unclear
Participants	23 intersections in Phoenix
Interventions	10 RLC intersections and 13 non‐camera comparison intersections in Phoenix. Comparisons selected on basis of similarity to treated intersections
Outcomes	Angle crashes, left turn crashes, rear end crashes
Notes	Crashes involving factors such as alcohol, drugs, illness or fatigue were removed. Target crashes within 30 m from intersection determined to be red light related. Results reported for target approaches and all approaches, data extracted for all approaches

Scottsdale, AZ—EB

**Table:**

Methods	CBA with Empirical Bayes. Data collected between Jan 1991 and December 2003. RLC installation dates and before/after periods unclear
Participants	14 intersections in Scottsdale
Interventions	14 RLC intersections
Outcomes	Angle crashes, left turn crashes, rear end crashes.
Notes	Crashes involving factors such as alcohol, drugs, illness or fatigue were removed. Target crashes within 30 m from intersection determined to be red light related. Results reported for target approaches and all approaches, data extracted for all approaches

Sohn and Bandini (2012; City of Las Cruces, New Mexico, USA)

**Table:**

Methods	CBA. Data collected between Jan 2004 and April 2011 with RLC program initiating in March 2009
Participants	10 intersections in City of Las Cruces, NM
Interventions	4 RLC intersections (cameras on multiple approaches) and 6 non‐camera intersections in Las Cruces
Outcomes	Total crashes, angle crashes, rear end crashes, PDO crashes, angle injury crashes, rear end injury crashes
Notes	

### Excluded studies

**Table:**

Study	Reason for exclusion
Blais and Carnis (2015)	RLCs not primary intervention of interest. Study examines the French Automated Speed Enforcement Program (ASEP)—cannot separate effect of RLCs from ASEP
Budd et al. (2011)	All intersections without RLCs located within the same postcode as a treatment sites
Dahnke et al. (2008)	No independent control group, non‐monitored approaches of RLC intersection used as controls
De Pauw et al. (2014)	Comparison group is database of all crashes in Flanders to control for trend, includes treated intersections
Golub et al. (2002)	No independent control group, used non‐monitored/unenforced approaches of RLC intersection as comparison.
Guerin (2010)	Speed cameras and RLCs activated at the same time ‐ cannot separate effect of RLCs from speed cameras.
Hebert‐Martinez and Porter (2006)	Focus on identifying and predicting characteristics of red light runner drivers after implementation of RLC program. Outcome measures not of interest.
Kent et al. (1995)	Ordinal logistic model ‐ not a CBA, no before data
Loftis et al. (2011)	No non‐RLC control intersection used. RLC intersections not selected at random. Study used simulation approach to model collisions with and without RLCs installed
Lum and Wong (2003a)	No independent control group—non‐monitored approaches of RLC intersection used as control. No examination of collision, only red light running. Study examines impact of RLCs on after‐red times—does RLC effect mean after‐red times on camera vs non‐camera approaches
Lum and Wong (2003b)	Same data as Lum and Wong (2003a), some of the same results reported. No independent control group—nonmonitored approaches of RLC intersection used as control
Lum and Wong (2003c)	Same data as Lum and Wong (2003a), some of the same results reported. No independent control group—nonmonitored approaches of RLC intersection used as control
McCartt and Hu (2014)	No before data. Only data during warning period at which time cameras and signs had been installed and public announcement made
McFadden and McGee (1999)	Report is a synthesis of other RLC studies. No new data provided
MVA Consultancy 1995	Some non‐camera approaches of RLC intersections used as controls (only 19 hr after monitoring)
Obeng and Burkey (2008)	Focus is on offsetting driver behavior—RLC intervention not the primary intervention of interest
Radalj (2001)	No control before after crash data. Excluded from previous review “no base data provided on control”
Shah (2010)	Not a controlled before after study. Mentions a report of a controlled before after study that was acquired but only reports percentage decreases and none of the data is included
Shah (2014)	Not a controlled before after study. Mentions a report of a control before after study that was acquired but only reports percentage decreases and none of the data is included
Smith et al. (2000)	Report is a synthesis of other RLC studies. No new data provided
Sun et al. (2012)	No before after comparison. Data collected using video‐based system set up by researchers. Intersections located 800 m apart (approximately 1/2 mile)
Synectics Transportation Consultants (2003; red light camera systems and stepped‐up police enforcement)	Study examines both RLCs and “stepped up police enforcement” (not clearly defined). Publicity campaign on RLR also conducted prior to and during the study. No signage used.
Synectics Transportation Consultants (2003; red light running evaluation)	Not clear whether data was collected on more than one day during each phase of the study. During the interim period, violation data were collected twice at the distant comparison sites but only once at the other locations
Vanlaar et al. (2014)	Study examines combined speed and RLC program—cannot separate effect of RLCs from speed cameras
Wang et al. (2015)	Study examines CMFs (crash modification factors), analyzed trends of CMF. RLCs not primary intervention of interest.
Walden (2011)	No independent control. Non‐red light crashes at treated intersections used as comparison group
Wong (2014)	Signal phasing changed concurrently with installation of RLCs so exclude as combined intervention
Yoo (2012)	No control group—location of study not specified

## APPENDIX G ASSESSMENT OF RISK OF BIAS

**Table:**

Dimension	Criteria
(1) Selection and matching of intervention and control areas	The characteristics of the study and control sites were the same/similar There were no changes in the control sites during the study period The control sites were not adjacent to the intervention sites
	It is unlikely that the control group received the intervention
(2) Blinding of data collection and analysis	The outcome data were obtained from routine reporting systems (not originally collected for study) The analysis was conducted blind to intervention and control groups
(3) Lengths of data collection time period pre‐ and post‐intervention	Length of before period is at least 1 year Length of after period is at least 1 year
(4) Reporting of results	The main findings of the study are clearly described The authors report uncertainty due to random variability (confidence intervals) Appropriate statistical tests were used to assess the main outcomes reported (*p*‐Values)
(5) Control of confounders	The authors describe potential confounders The distributions of confounders in intervention and control sites were assessed and similar Do the authors discuss the effect of confounders on the results?
(6) Any other potential sources of bias	Did the study control for potential bias due to regression to the mean? Did the study report, or control for “spill‐over” effects (e.g., use control sites located away from red light camera sites and associated publicity)? Were any other sources of bias addressed?
